# Supplementary figures and images for: Liver gene expression and its rewiring in hepatic steatosis are controlled by PI3Kα-dependent hepatocyte signaling
Source: PLoS Biol. 2025 Apr 14;23(4):e3003112. doi: 10.1371/journal.pbio.3003112 (PMC12021288; doi:10.1371/journal.pbio.3003112)

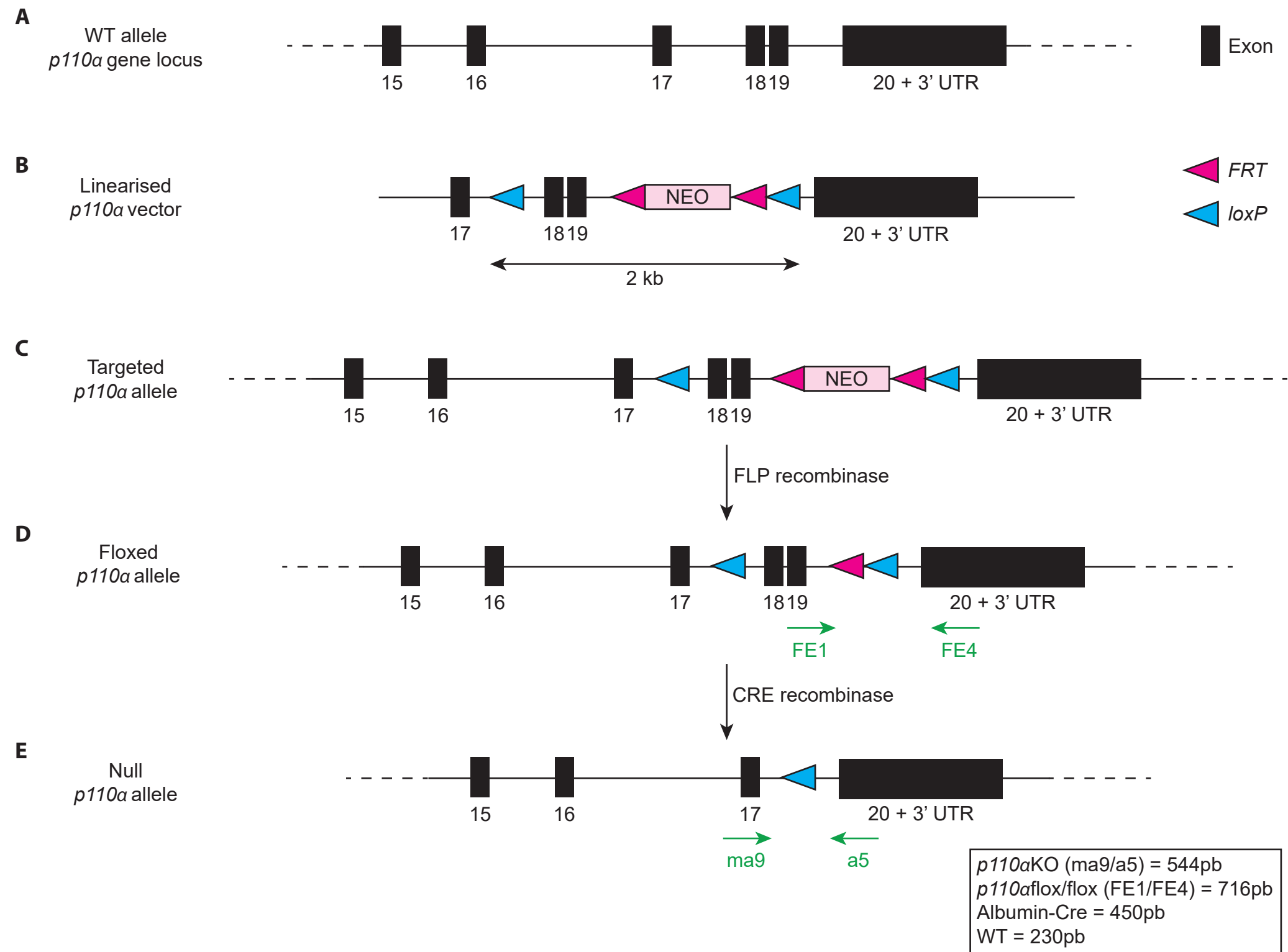

Supplement: S1 Fig — (A) Organization of the p110α targeted locus. (B) p110α targeting vector including the loxP sites surrounding exons 18 and 19 (length = 2 kb) and the PGK/neomycin selection cassette flanked by FRT sites and inserted between exon 19 and the second loxP site. (C) Targeted p110α allele containing loxP sites, neomycin resistance selection cassette, and FRT sites. (D) Targeted p110α allele following FLP recombinase-mediated deletion of the neomycin resistance selection cassette. (E) Targeted p110α allele following CRE-mediated deletion of floxed exons 18 and 19. Intron sequences are represented by a black line. Exon sequences are represented by filled black rectangles. The loxP and FRT sequences are represented by blue and pink triangles, respectively. The position of the primers used to validate p110α floxed and deleted alleles by PCR are represented by green arrows. The length of the different targeted DNA fragments is written below the schematic construction. (PDF) [file pbio.3003112.s001.pdf]

A

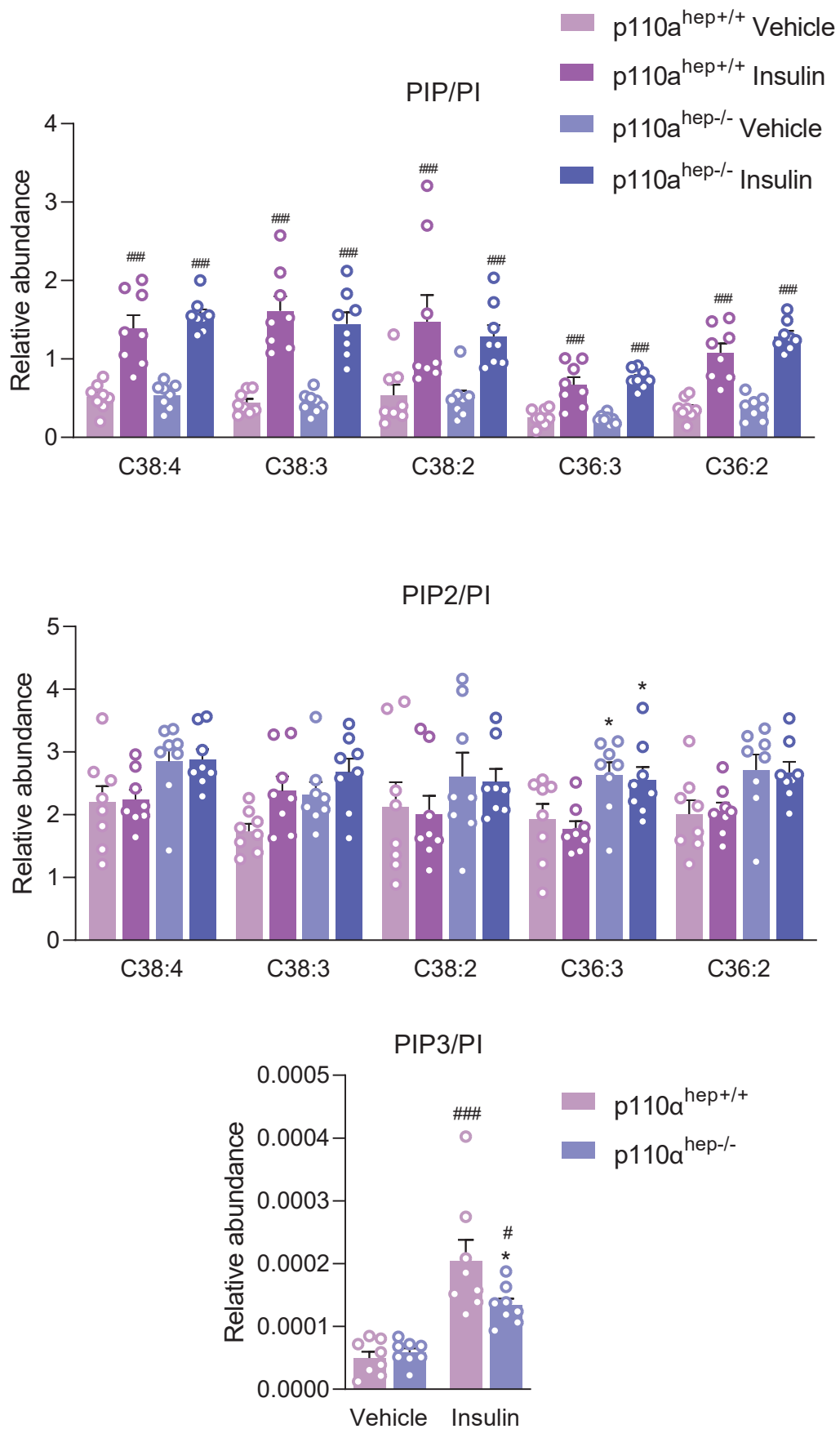

B

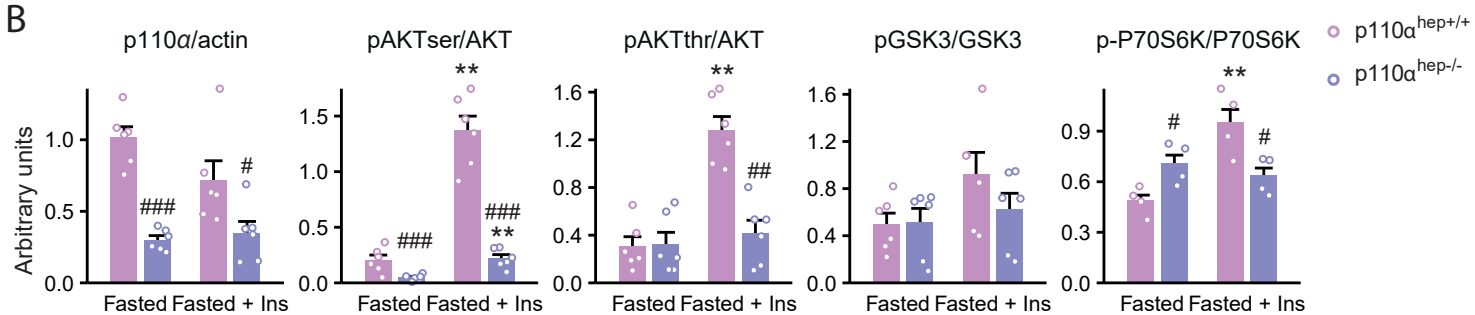

Supplement: S2 Fig — (A) Phosphatidylinositol phosphate (PIP), and phosphatidylinositol biphosphate (PIP2) and phosphatidylinositol triphosphate (PIP3) relative abundance in liver samples from p110αhep+/+ and p110αhep−/− mice after fasting or under fasted conditions and treated with insulin (5 U/kg) through inferior vena cava injection (n = 7–8 mice/genotype/experimental condition). The analysis was performed by mass spectrometry and the relative abundance for each molecular species were calculated as a ratio to Phosphatidylinositol (PI). This method yields the number of phosphorylations and the fatty-acyl compositions of phosphatidylinositol. For example, PIP3 (C38:4) means that the phosphatidylinositol lipid has a mass that corresponds to three phosphorylations of the inositol and contains acyl chains with 38 carbons and 4 double bonds. (B) Quantification of signals from immunoblots, represented in Fig 1C. The numerical values underlying the panels for this figure can be found in S6 Data. (PDF) [file pbio.3003112.s002.pdf]

**A**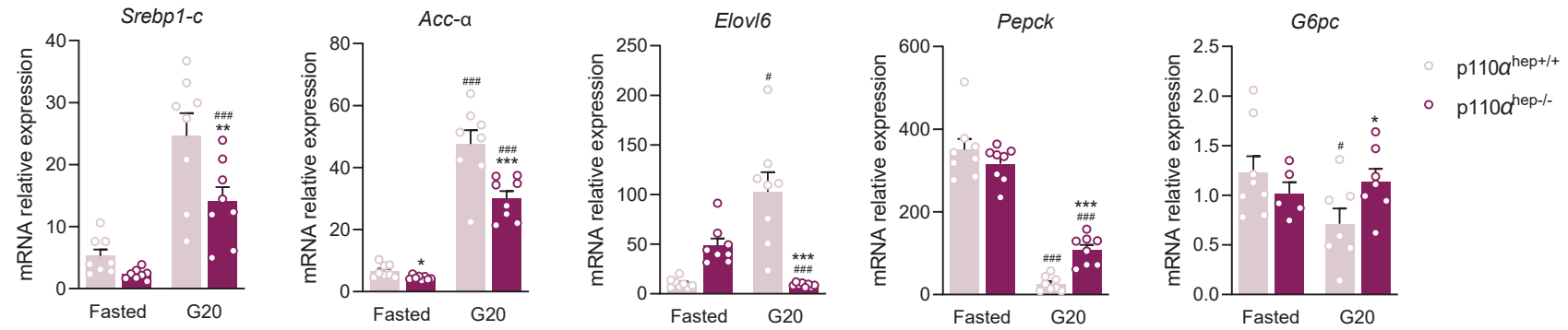**B**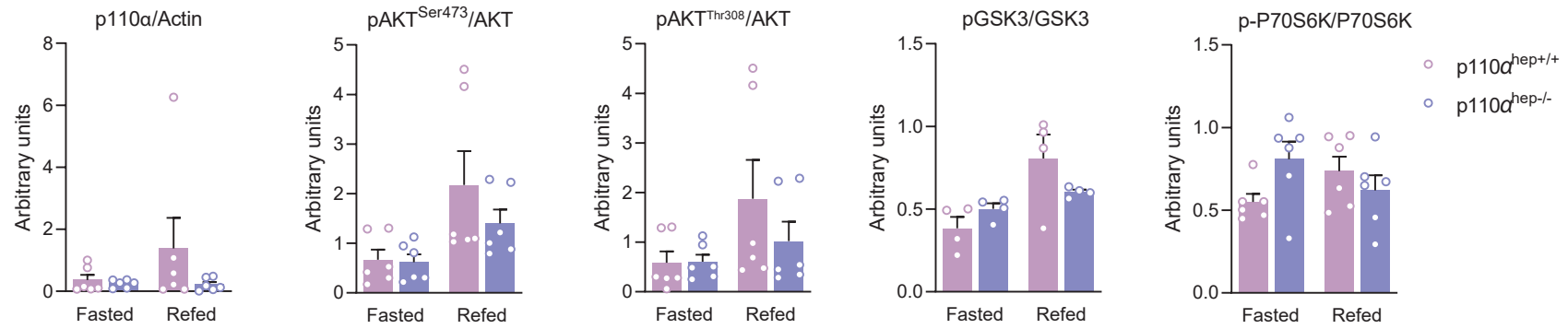**C**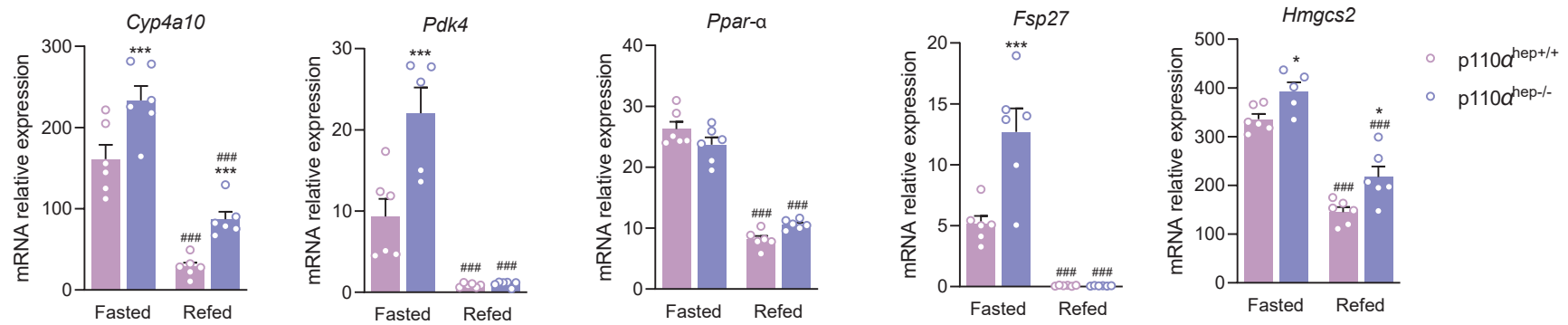

Supplement: S4 Fig — (A) Relative expression of Srebp1-c, Acc-α, Elovl6, Pepck, and G6pc mRNA in liver tissue from p110αhep+/+ and p110αhep−/− mice under fasting conditions or supplemented with 20% glucose in drinking water (n = 8 mice/genotype/experimental condition). (B) Quantification of signals from immunoblots, represented in Fig 3F. (C) Expression of Cyp4a10, Pdk4, Pparα, Fsp27, and Hmgcs2 mRNA in liver tissue from p110αhep+/+ and p110αhep−/− mice under fasting conditions or supplemented with 20% glucose in drinking water (n = 8 mice/genotype/experimental condition). The numerical values underlying the panels for this figure can be found in S8 Data. (PDF) [file pbio.3003112.s004.pdf]

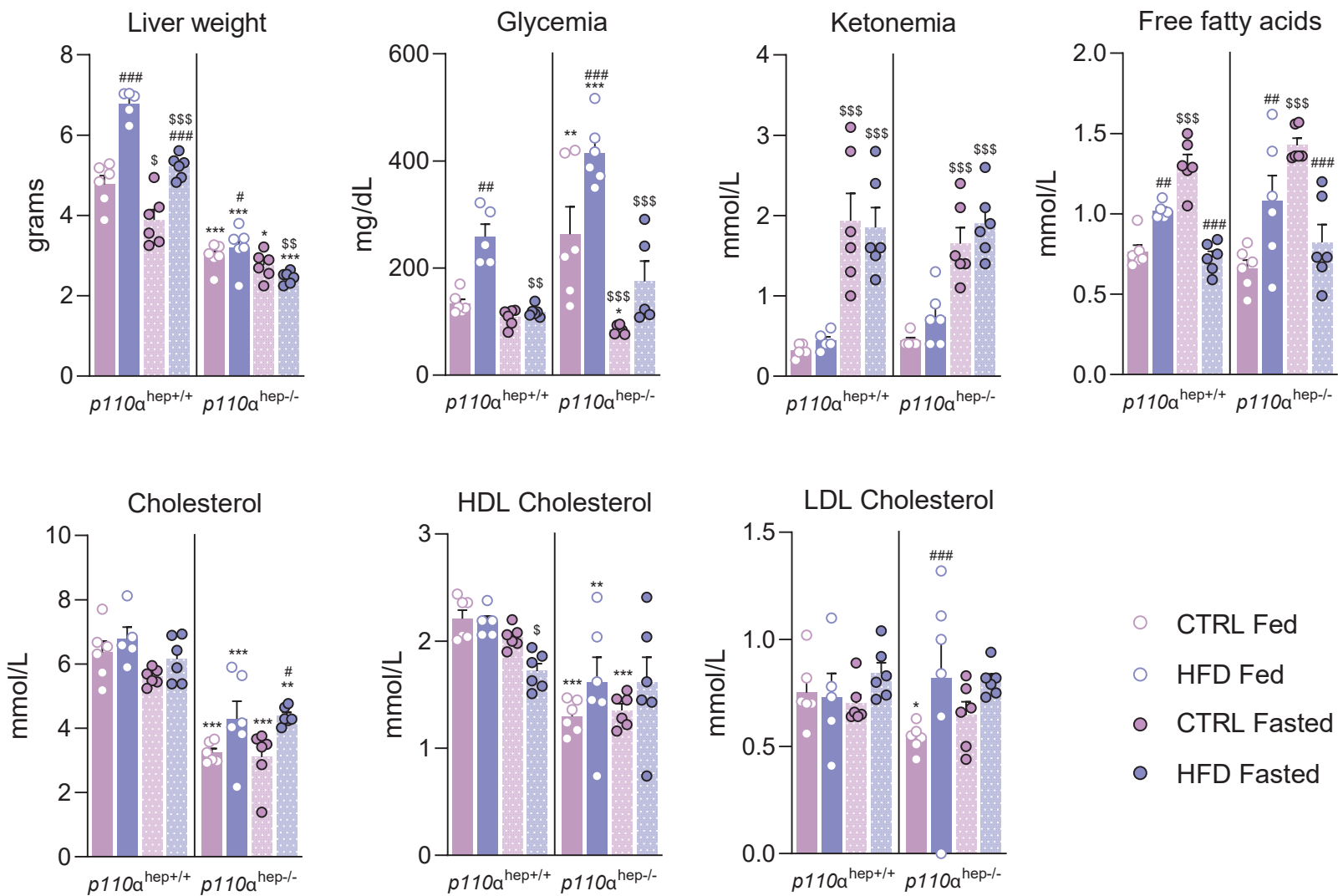

Supplement: S5 Fig — Liver weight and plasma measurements including glucose levels, ketone bodies, free fatty acids, and cholesterol levels (total, HDL, and LDL). Data are presented as mean ± SEM (n = 6 mice per group). #P ≤ 0.05, ##P ≤ 0.01, and ###P ≤ 0.005 for the effect of the diet (CTRL versus HFD); $P ≤ 0.05, $$P ≤ 0.01, and $$$P ≤ 0.005 for the nutritional status (fed versus fasted); *P ≤ 0.05, **P ≤ 0.01, and ***P ≤ 0.005 for genotype effect (+/+ versus −/−). The numerical values underlying the panels for this figure can be found in S9 Data. (PDF) [file pbio.3003112.s005.pdf]

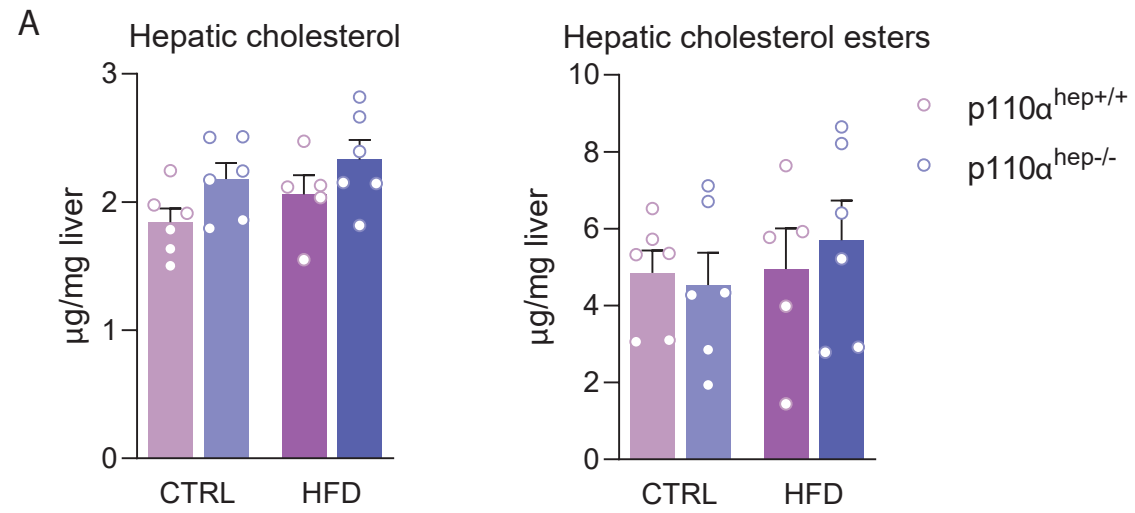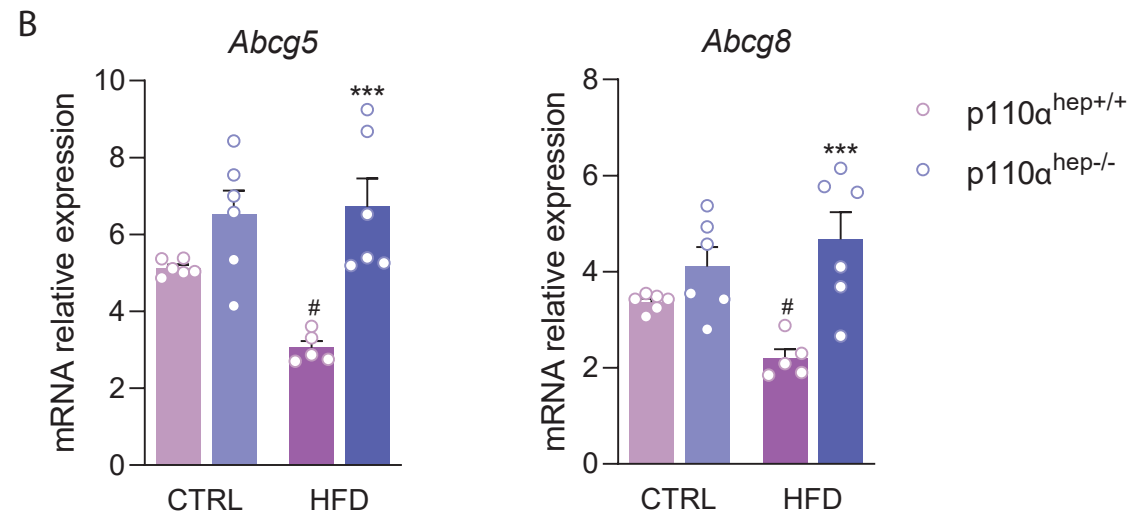

Supplement: S6 Fig — . (A) Hepatic cholesterol and cholesterol esters levels in p110αhep+/+ and p110αhep−/− mice fed a CTRL diet or HFD. (B) Expression of Abcg5 and Abcg8 mRNA in liver tissue from p110αhep+/+ and p110αhep−/− mice fed a CTRL diet or HFD. The numerical values underlying the panels for this figure can be found in S10 Data. (PDF) [file pbio.3003112.s006.pdf]

A

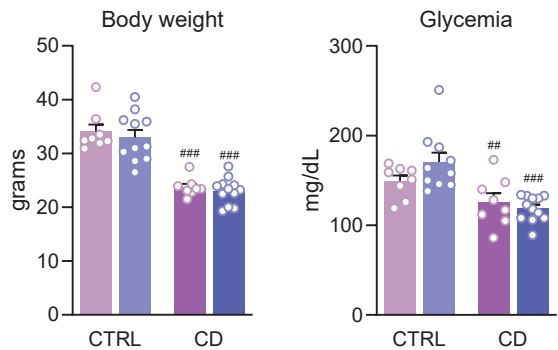

B

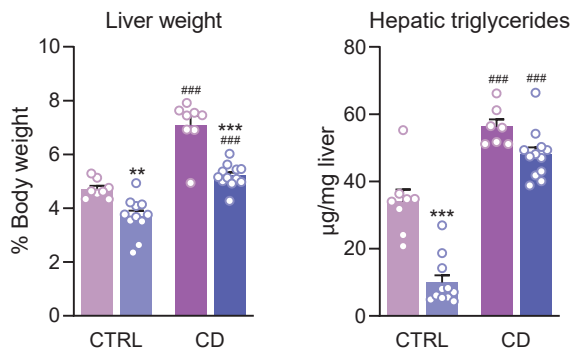

C

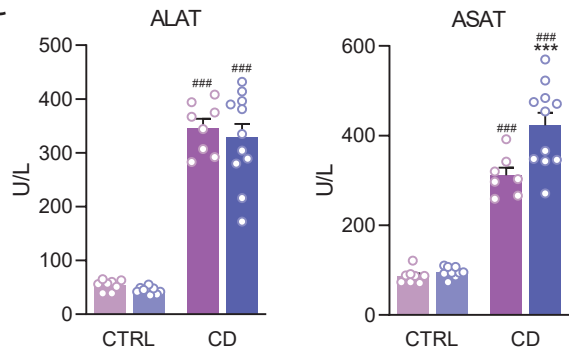

D

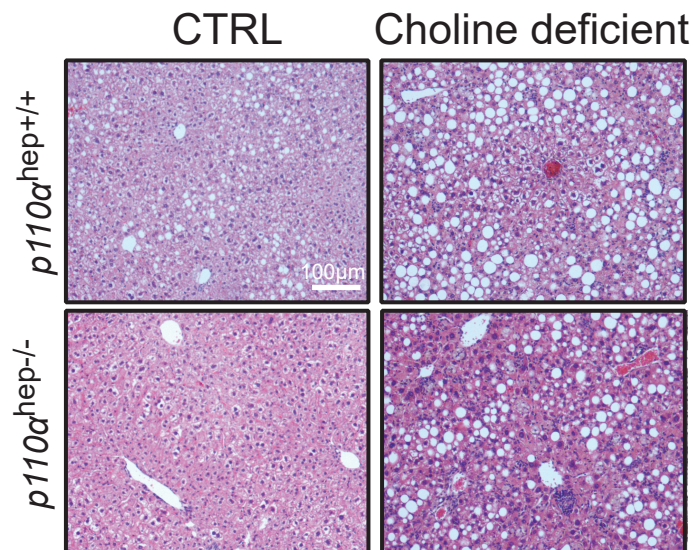

■  $p110\alpha^{hep+/+}$  CTRL  
 ■  $p110\alpha^{hep-/-}$  CTRL  
 ■  $p110\alpha^{hep+/+}$  CD  
 ■  $p110\alpha^{hep-/-}$  CD

Supplement: S7 Fig — (A) Body weight and plasma glucose levels in the mice from (A) in 12-week-old p110αhep+/+ and p110αhep−/− mice fed a chow diet (CTRL) or a choline-deficient HFD (CD-HFD) for 12 weeks (n = 6/genotype). (B) Relative liver weight and liver triglyceride content in the mice from (A). (C) Plasma ALT and AST activity. (D) Representative pictures of H/E staining of liver sections. Scale bar, 100 µm. Data information: In all graphs, data are presented as mean ± SEM. #P ≤ 0.05, ##P ≤ 0.01, and ###P ≤ 0.005 for diet effect; *P ≤ 0.05, **P ≤ 0.01, and ***P ≤ 0.005 for genotype effect. The numerical values underlying the panels for this figure can be found in S11 Data. (PDF) [file pbio.3003112.s007.pdf]

## Hepatokines

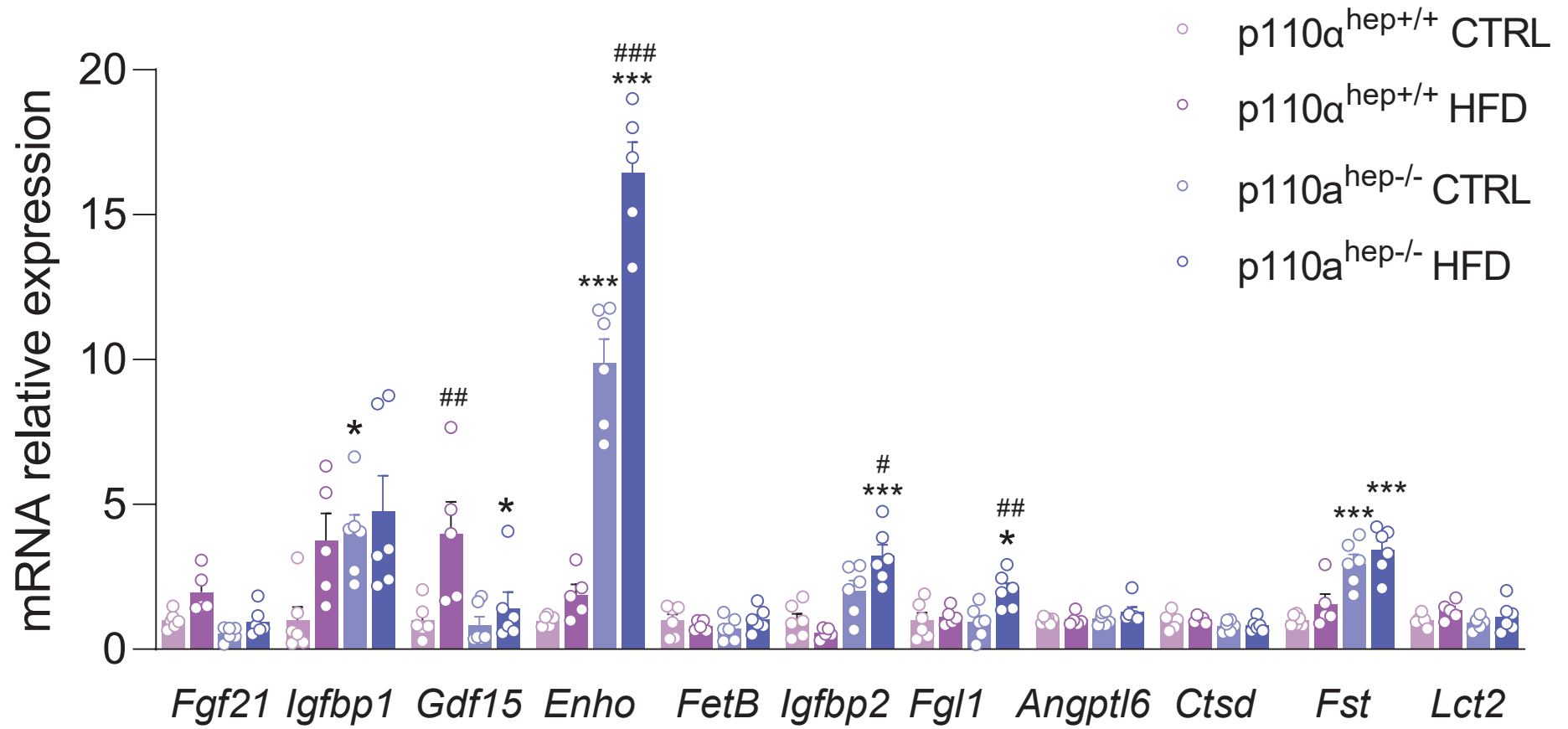

Supplement: S9 Fig — The relative expression of Fgf21, Igfbp1, Gdf1, Enho, FetB, Igfbp2, Fgl1, Angptl6, Ctsd, Fst, and Lect2 mRNA in liver extracts from p110αhep+/+ and p110αhep−/− mice fed a CTRL diet or HFD (n = 6 mice/group/experimental condition). The numerical values underlying the panels for this figure can be found in S13 Data. (PDF) [file pbio.3003112.s009.pdf]

## Glycogen

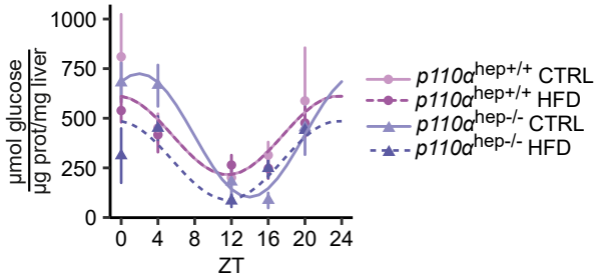

Supplement: S10 Fig — Quantification of liver glycogen content of HFD-fed p110αhep+/+ and p110αhep−/− mice over 5 ZTs (ZT0, ZT4, ZT12, ZT16 and ZT20). Results were normalized to the quantity of proteins for each mouse. The numerical values underlying the panels for this figure can be found in S14 Data. (PDF) [file pbio.3003112.s010.pdf]
