# Supplementary material for: Liver gene expression and its rewiring in hepatic steatosis are controlled by PI3Kα-dependent hepatocyte signaling
Source: PLoS Biol. 2025 Apr 14;23(4):e3003112. doi: 10.1371/journal.pbio.3003112 (PMC12021288; doi:10.1371/journal.pbio.3003112)
Supplement: S3 Fig — (A) Enrichment analysis of the genes significantly down-regulated (left) and up-regulated (right) between p110αhep+/+ and p110 αhep−/− under fed conditions. (B) Left: Coefficient plots related to the O-PLS-DA models from 1H-nuclear magnetic resonance (NMR) discriminating between p110αhep+/+ and p110αhep−/− mice under fed conditions. The figure shows the discriminant metabolites that are higher or lower in p110αhep+/+ versus p110αhep−/− mice. Metabolites are color-coded according to their correlation coefficient, with red indicating a very strong positive correlation (R2 > 0.65). The direction of the metabolite indicates the group with which it is positively associated, as labeled on the diagram. Right: Area under the curve of the 1H-NMR spectra was integrated for the lactate and betaine signals. (C) Plasma levels of triglycerides and total, HDL, and LDL cholesterol in p110αhep+/+ and p110αhep−/− mice under fed or fasted conditions (n = 6 mice/group/experimental condition). The numerical values underlying the panels for this figure can be found in S7 Data. (PDF) [file pbio.3003112.s003.pdf]

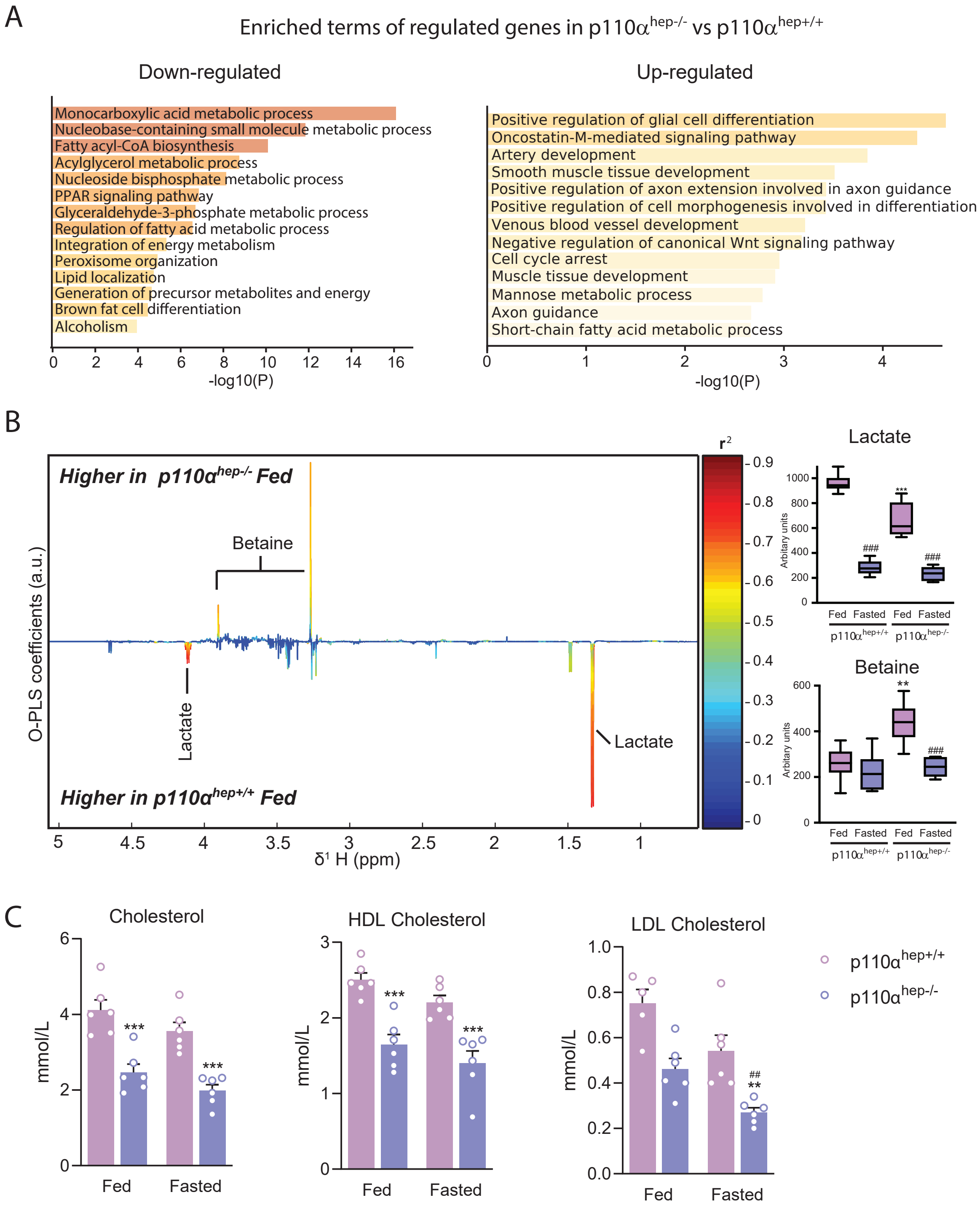

A
Enriched terms of regulated genes in p110α hep-/- vs p110α hep+/+
Down-regulated
Monocarboxylic acid metabolic process
Nucleobase-containing small molecule metabolic process
Fatty acyl-CoA biosynthesis
Acylglycerol metabolic process
Nucleoside bisphosphate metabolic process
PPAR signaling pathway
Glyceraldehyde-3-phosphate metabolic process
Regulation of fatty acid metabolic process
Integration of energy metabolism
Peroxisome organization
Lipid localization
Generation of precursor metabolites and energy
Brown fat cell differentiation
Alcoholism
-log10(P)
Up-regulated
Positive regulation of glial cell differentiation
Oncostatin-M-mediated signaling pathway
Artery development
Smooth muscle tissue development
Positive regulation of axon extension involved in axon guidance
Positive regulation of cell morphogenesis involved in differentiation
Venous blood vessel development
Negative regulation of canonical Wnt signaling pathway
Cell cycle arrest
Muscle tissue development
Mannose metabolic process
Axon guidance
Short-chain fatty acid metabolic process
-log10(P)
B
Higher in p110α hep-/- Fed
Betaine
Lactate
Lactate
Higher in p110α hep+/+ Fed
O-PLS coefficients (a.u.)
δ¹ H (ppm)
r²
Lactate
Arbitary units
Fed Fasted Fed Fasted
p110α hep+/+ p110α hep-/-
Betaine
Arbitary units
Fed Fasted Fed Fasted
p110α hep+/+ p110α hep-/-
C
Cholesterol
HDL Cholesterol
LDL Cholesterol
mmol/L
Fed Fasted
p110α hep+/+
p110α hep-/-
