# Supplementary material for: Liver gene expression and its rewiring in hepatic steatosis are controlled by PI3Kα-dependent hepatocyte signaling
Source: PLoS Biol. 2025 Apr 14;23(4):e3003112. doi: 10.1371/journal.pbio.3003112 (PMC12021288; doi:10.1371/journal.pbio.3003112)
Supplement: S8 Fig — (A) Heatmap of hepatic lipid quantification in liver samples (n = 6/group). Hierarchical clustering highlights seven different clusters based on the lipid levels. (B) Relative abundance of phosphatidyl ethanolamine (PE) 40:3 and phosphatidyl choline (PC) 36:3 (n = 6/genotype/experimental condition). (C) Relative abundance of PC 32:1 and triglyceride (TG) C51 (n = 6/genotype/experimental condition). (D) Relative abundance of TG C55 and ceramide (Cer) C18:0 (n = 6/genotype/experimental condition). (E) Relative abundance of PE 38:4 and Cer C16:0 (n = 6/genotype/experimental condition). (F) Relative abundance of esterified cholesterol C18 and PE 36:1 (n = 6/genotype/experimental condition). (G) Relative abundance of fatty acid C20:5n-3 and PI 36:1. (H) Differentially expressed genes involved in lipid trafficking, fatty acid metabolism, unsaturated fatty acid metabolism, lipid droplets, cholesterol metabolism, phospholipid metabolism, sphingolipid metabolism, and cannabinoid metabolism between p110αhep+/+ and p110αhep−/− in mice fed CTRL diet (yellow) and HFD (grey), respectively. Data information: In all graphs, data are presented as mean ± SEM. #P ≤ 0.05 for treatment effect, *P ≤ 0.05 for genotype effect. The numerical values underlying the panels for this figure can be found in S12 Data. (PDF) [file pbio.3003112.s008.pdf]

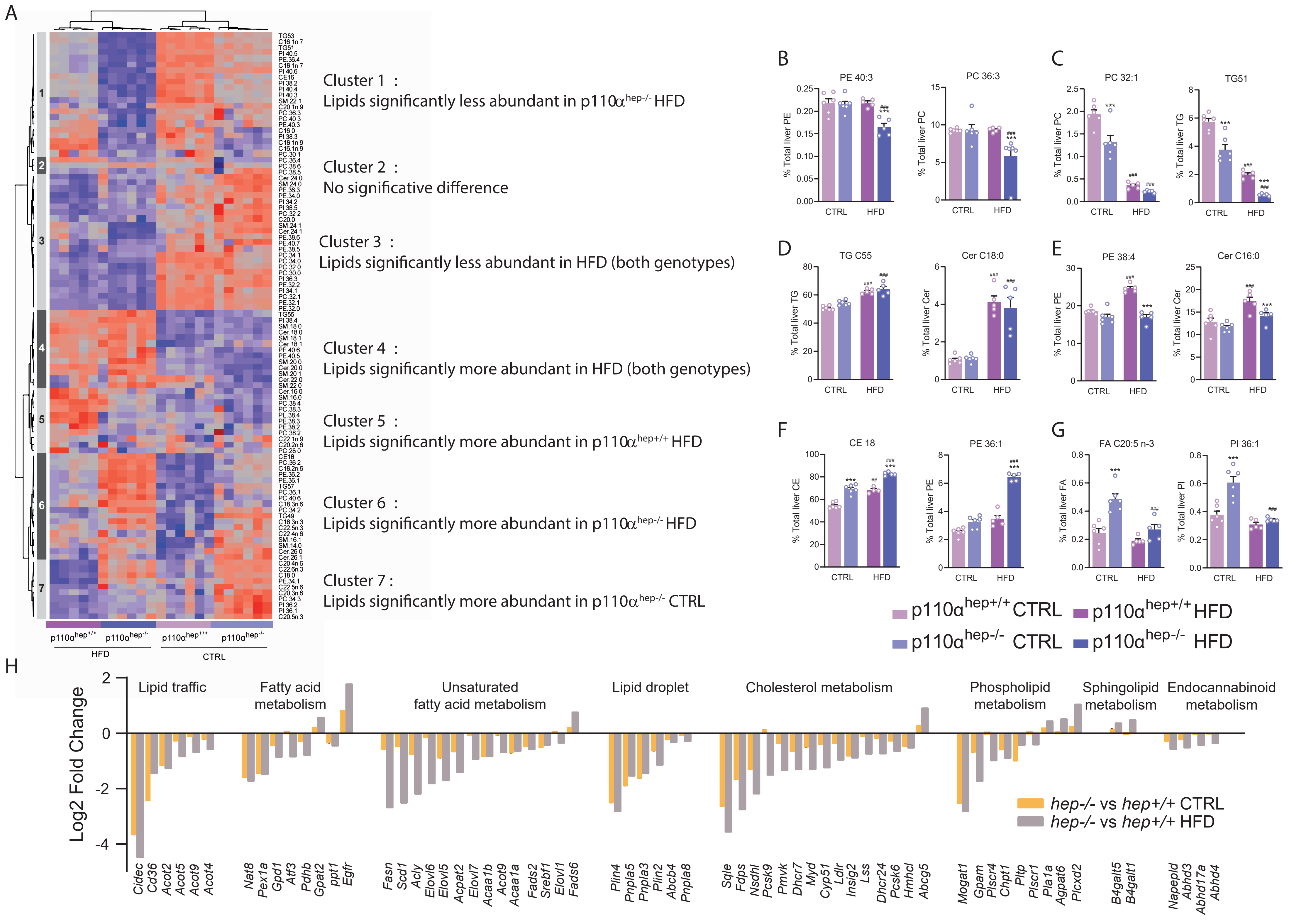

A
Cluster 1 : Lipids significantly less abundant in p110α hep-/- HFD
Cluster 2 : No significative difference
Cluster 3 : Lipids significantly less abundant in HFD (both genotypes)
Cluster 4 : Lipids significantly more abundant in HFD (both genotypes)
Cluster 5 : Lipids significantly more abundant in p110α hep+/+ HFD
Cluster 6 : Lipids significantly more abundant in p110α hep-/- HFD
Cluster 7 : Lipids significantly more abundant in p110α hep-/- CTRL
p110α hep+/+
p110α hep-/-
HFD
CTRL
B
PE 40:3
PC 36:3
C
PC 32:1
TG51
D
TG C55
Cer C18:0
E
PE 38:4
Cer C16:0
F
CE 18
PE 36:1
G
FA C20:5 n-3
PI 36:1
% Total liver PE
% Total liver PC
% Total liver TG
% Total liver Cer
% Total liver CE
% Total liver FA
% Total liver PI
CTRL
HFD
p110α hep+/+ CTRL
p110α hep+/+ HFD
p110α hep-/- CTRL
p110α hep-/- HFD
H
Log2 Fold Change
Lipid traffic
Fatty acid metabolism
Unsaturated fatty acid metabolism
Lipid droplet
Cholesterol metabolism
Phospholipid metabolism
Sphingolipid metabolism
Endocannabinoid metabolism
hep-/- vs hep+/+ CTRL
hep-/- vs hep+/+ HFD
Cidec Cd36 Acot2 Acot5 Acot9 Acot4
Nat8 Pex1a Gpd1 Atf3 Pdhb Gpat2 ppt1 Egfr
Fasn Scd1 Acly Elovl6 Elovl5 Acpat2 Elovl7 Acaa1b Acot9 Acaa1a Fads2 Srebf1 Elovl1 Fads6
Plin4 Pnpla5 Pnpla3 Plin2 Abcb4 Pnpla8
Sqle Fdps Nsdhl Pcsk9 Pmvk Dhcr7 Myd Cyp51 Ldlr Insig2 Lss Dhcr24 Pcsk6 Hmhcl Abcg5
Mogat1 Gpam Plscr4 Chpt1 Pltp Plscr1 Pla1a Agpat6 Plcxd2
B4galt5 B4galt1
Napepld Abhd3 Abhd17a Abhd4
