# Supplementary material for: Liver gene expression and its rewiring in hepatic steatosis are controlled by PI3Kα-dependent hepatocyte signaling
Source: PLoS Biol. 2025 Apr 14;23(4):e3003112. doi: 10.1371/journal.pbio.3003112 (PMC12021288; doi:10.1371/journal.pbio.3003112)
Supplement: S1 Table — List of primers used for real-time quantitative polymerase chain reaction (qPCR) analyses. (PDF) [file pbio.3003112.s011.pdf]

S1 Table – List of oligonucleotide sequences used for real-time qPCR

Gene	NCBI Refseq	Forward primer (5'-3')	Reverse primer (5'-3')
Acaca	NM_133360	TTACAGGATGGTTTGGCCTTTC	CAAATTCTGCTGGAGAAGCCAC
Acly	NM_134037	AAAGCTTGGCCTCGTCGG	GGGACGAAGGGTTCAATGAGA
Angptl6	*NM_145154*	*GAATTGCCGCAAACCTCACT*	*ATGGCCGTCACCTCTCACAG*
Chrebpα	NM_021455	CGACACTCACCCACCTCTTC	TTGTTCAGCCGGATCTTGTC
Chrebpβ	NM_021455	TCTGCAGATCGCGTGGAG	CTTGTCCCGGCATAGCAAC
Ctsd	*NM_009983*	*CTTCGTCCTCCTTCGCGATTAT*	*GTCCGACGGATAGATGTGAACTT*
Cyp4a10	*NM_010011*	*TCCAGCAGTTCCCATCACCT*	*TTGCTTCCCCAGAACCATCT*
Cyp4a14	NM_007822	TCAGTCTATTTCTGGTGCTGTTC	GAGCTCCTTGTCCTTCAGATGGT
Enho	NM_027147	ACCGGGCTCAACTCAGGC	TGGCTGTCCTGTCCACACAC
Elovl6	NM_130450	TCTGATGAACAAGCGAGCCA	TGGTCATCAGAATGTACAGCATGT
Fasn	NM_007988	AGTCAGCTATGAAGCAATTGTGGA	CACCCAGACGCCAGTGTTC
Fetuinb	*NM_021564*	*CTCGTCAAAGTCACCAAGGCTAT*	*CACATAGTAAGCAGGGCCAGAC*
Fgf21	NM_020013	AAAGCCTCTAGGTTTCTTTGCCA	CCTCAGGATCAAAGTGAGGCG
Fgl1	*NM_145594*	*TGCAAACCTGAACGGTGTTTAC*	*TTCAAGGAATACCACCACCCA*
Fsp27	NM_178373	AGGCCCTGTCGTGTTAGCAC	CATGATGCCTTTGCGAACCT
Fst	*NM_008046*	*TGCTGCTACTCTGCCAGTTCAT*	*CACTCTTCCTTGCTCAGTTCTGTC*
Gck	NM_010191	AGGAGTTCTATAACCTGCTGCGAT	GGTCTTCAAGCTGCTGCTGAGT
Gdf15	*NM_011819*	*GCTGTCCGGATACTCAGTCCA*	*TTGACGCGGAGTAGCAGCT*
G6pc	NM_008061	CTCACTTTCCCCACCAGGTC	GCTGAAAGTTTCAGCCACAGC
Hmgcs2	NM_008256	TGCAGGAAACTTCGCTCACA	AAATAGACCTCCAGGGCAAGGA
Igfbp1	*NM_008341*	*CCTGCCAACGAGAACTCTAT*	*AGGGATTTTCTTTCCACTCC*
Igfbp2	*NM_008342*	*GCATGGCCGGTACAACCTTA*	*GCTGTCCGTTCAGAGACATCTT*
Lect2	*NM_010702*	*GTGGACAGTACTCTGCTCAAA*	*TCCCAGTGAATGGTGCATAC*
Lpk	*NM_013631*	*TCGACTCAGAGCCTGTGGC*	*AGTCGTGCAATGTTCATCCCT*
Pdk4	NM_013743	ATCGCCAGAATTAAACCTCACAC	TGGATTGGTTGGCCTGGA
Pepck	NM_011044	GAACCCCAGCCTGCCC	GAGCAACTCCAAAAAACCCG
Pnpla3	NM_054088	ACGCGGTCACCTTCGTGT	AGCCCGTCTCTGATGCACTT
Ppara	NM_011144	CCCTGTTTGTGGCTGCTATAATTT	GGGAAGAGGAAGGTGTCATCTG
Scd1	NM_009127	CAGTGCCGCGCATCTCTAT	CAGCGGTACTCACTGGCAGA
Srebp1-c	NM_011480	GGAGCCATGGATTGCACATT	GCTTCCAGAGAGGAGGCCAG
Vnn1	NM_011704	ATGAGGTTTATGCCTTTGGAGC	CCACAGGTGCGTAAATTGGTAG
