# Supplementary material for: Liver gene expression and its rewiring in hepatic steatosis are controlled by PI3Kα-dependent hepatocyte signaling
Source: PLoS Biol. 2025 Apr 14;23(4):e3003112. doi: 10.1371/journal.pbio.3003112 (PMC12021288; doi:10.1371/journal.pbio.3003112)

Annotated original image for agarose gel in Figure 1A

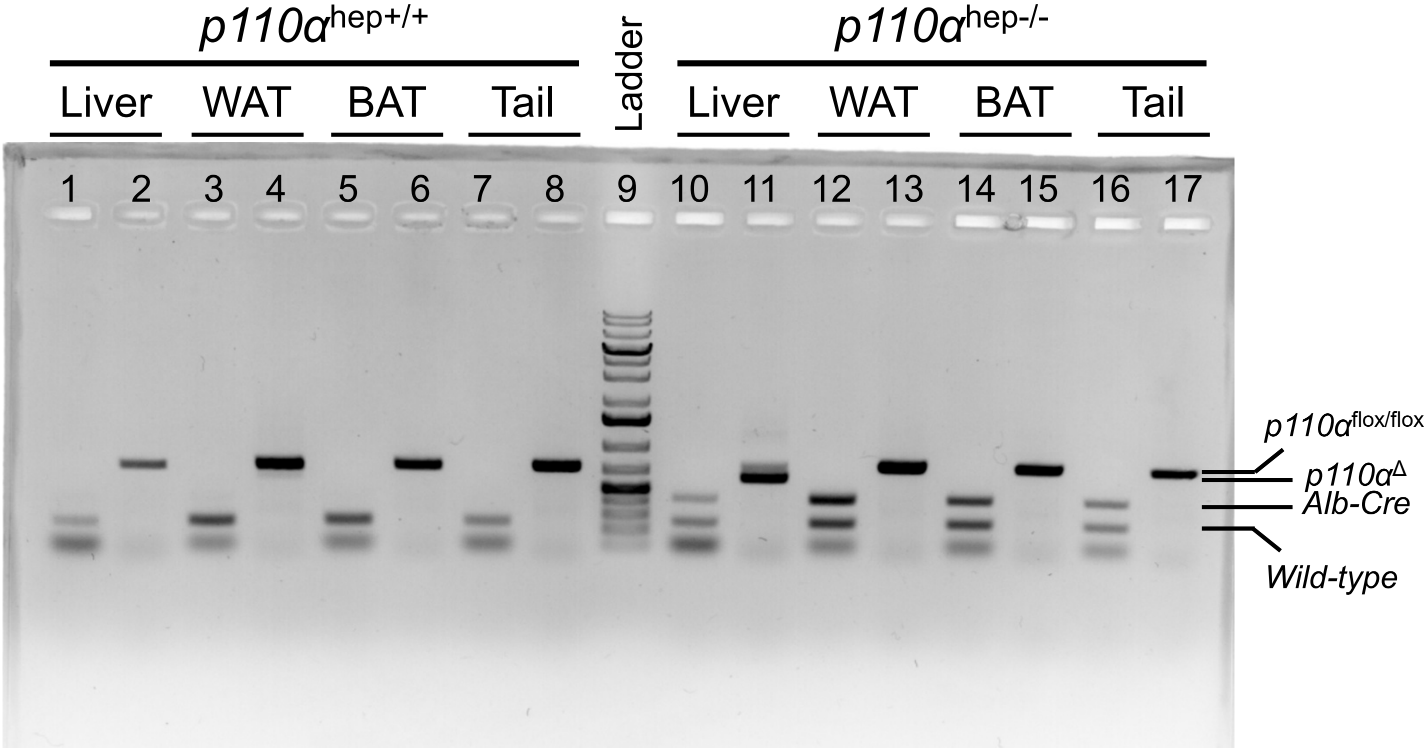

Lines 1,3,5,7,10,12,14,16 : PCR for detection of Albumin-Cre (Alb-Cre : 450 pb)
Lines 2,4,6,8,11,13,15,17 : PCR for detection of *p110α* floxed (*p110α* $^{flox/flox}$: 714 pb*)* and *p110α* deleted (*p110α*^{Δ} : 544 pb)

Annotated original images for western blots in Figure 1C

- Serie 1 : 9 samples/gel, n=2-3/experimental group

Samples 9, 14, 23, 24, 36, 38, 42, 45, 41

- Serie 2 : 8 samples/gel, n=2/experimental group

Samples 11, 12, 17, 18, 33, 37, 43, 48

→ In total n=4-5/experimental group

n° sample	genotype	insulin
9	p110α$^{hep+/+}$	-
11	p110α$^{hep+/+}$	-
12	p110α$^{hep+/+}$	-
14	p110α$^{hep+/+}$	-
17	p110α$^{hep+/+}$	+
18	p110α$^{hep+/+}$	+
23	p110α$^{hep+/+}$	+
24	p110α$^{hep+/+}$	+
33	p110α$^{hep-/-}$	-
36	p110α$^{hep-/-}$	-
37	p110α$^{hep-/-}$	-
38	p110α$^{hep-/-}$	-
41	p110α$^{hep-/-}$	+
42	p110α$^{hep-/-}$	+
43	p110α$^{hep-/-}$	+
45	p110α$^{hep-/-}$	+
48	p110α$^{hep-/-}$	+

<u>Serie 1</u> : 9 samples/gel, n=2-3/experimental group
Samples 9, 14, 23, 24, 36, 38, 42, 45, 41

<u>Primary antibody</u>: PI3Kinase p110α (1/1000 in 5% milk, 1X TBS, 0.1% Tween20 overnight at 4°C)
<u>Secondary antibody</u>: goat anti-rabbit IgG, HRP-linked (1/5000 in 1X TBS, 0.1% Tween20 1h at RT)

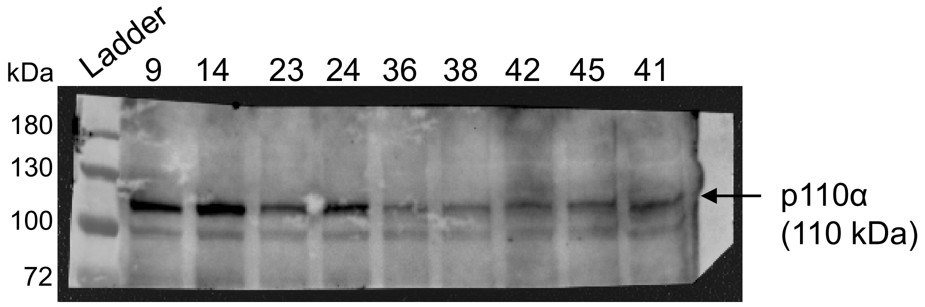

<u>Primary antibody</u>: Phospho-Akt (Ser473) (1/1000 in 5% BSA, 1X TBS, 0.1% Tween20 overnight at 4°C)
<u>Secondary antibody</u>: goat anti-rabbit IgG, HRP-linked (1/5000 in 1X TBS, 0.1% Tween20 1h at RT)

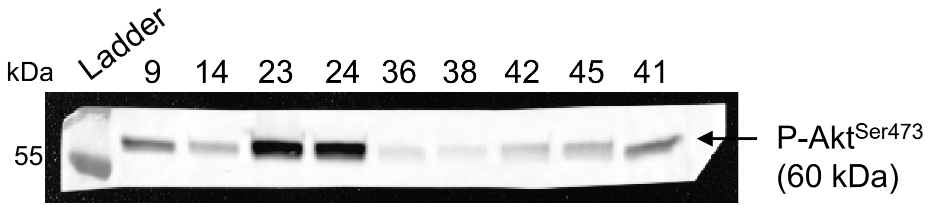

<u>Primary antibody</u>: Phospho-Akt (Thr308) (1/1000 in 5% BSA, 1X TBS, 0.1% Tween20 overnight at 4°C)
<u>Secondary antibody</u>: goat anti-rabbit IgG, HRP-linked (1/5000 in 1X TBS, 0.1% Tween20 1h at RT)

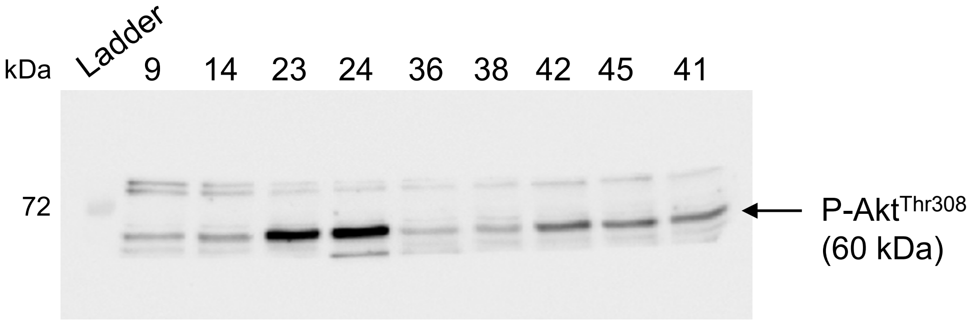

Serie 1 : 9 samples/gel, n=2-3/experimental group
Samples 9, 14, 23, 24, 36, 38, 42, 45, 41

Primary antibody: Akt (pan) (1/1000 in 5% BSA, 1X TBS, 0.1% Tween20 overnight at 4°C)
Secondary antibody: goat anti-rabbit IgG, HRP-linked (1/5000 in 1X TBS, 0.1% Tween20 1h at RT)

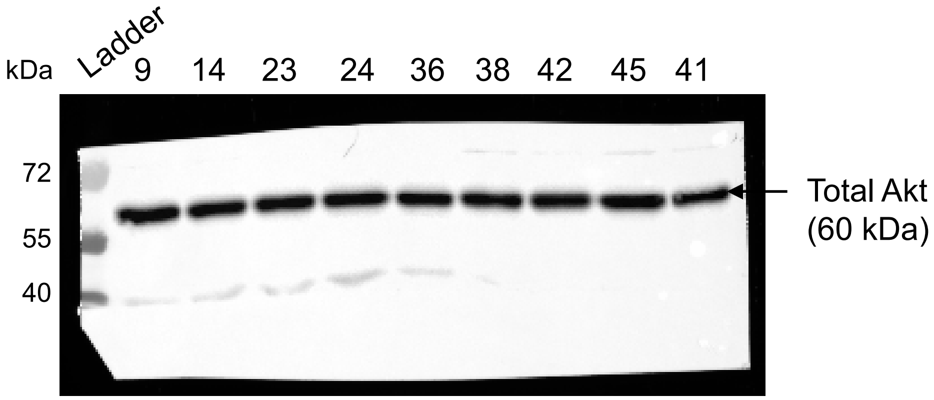

Primary antibody: Phospho- GSK-3β (Ser9) (1/1000 in 5% BSA, 1X TBS, 0.1% Tween20 overnight at 4°C)
Secondary antibody: goat anti-rabbit IgG, HRP-linked (1/5000 in 1X TBS, 0.1% Tween20 1h at RT)

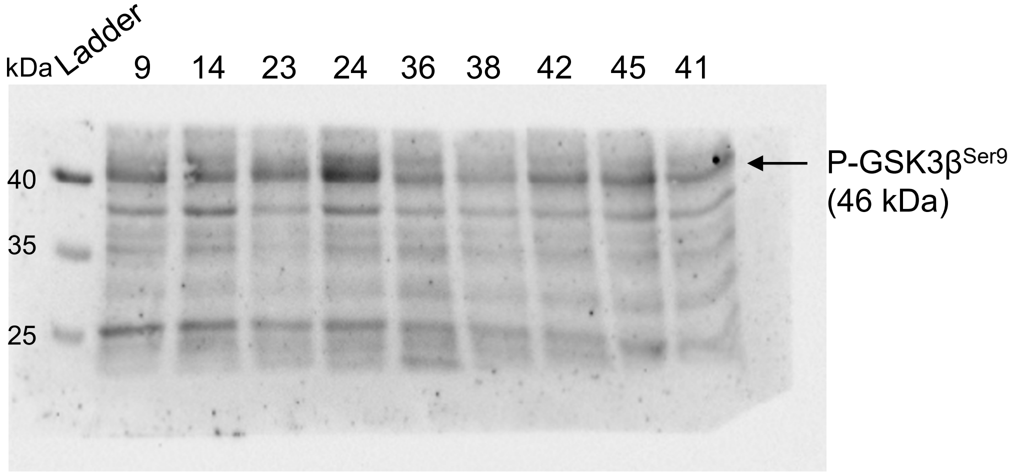

Primary antibody: GSK-3β (1/1000 in 5% BSA, 1X TBS, 0.1% Tween20 overnight at 4°C)
Secondary antibody: goat anti-rabbit IgG, HRP-linked (1/5000 in 1X TBS, 0.1% Tween20 1h at RT)

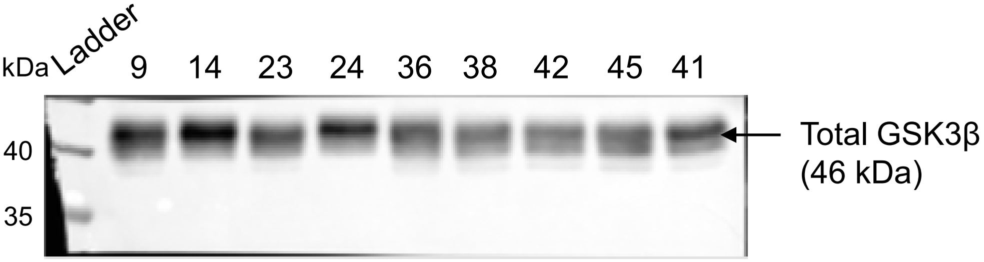

Serie 1 : 9 samples/gel, n=2-3/experimental group
Samples 9, 14, 23, 24, 36, 38, 42, 45, 41

Primary antibody: Phospho-p70S6kinase (Thr389) (1/1000 in 5% BSA, 1X TBS, 0.1% Tween overnight at 4°C)
Secondary antibody: goat anti-rabbit IgG, HRP-linked (1/5000 in 1X TBS, 0.1% Tween20 1h at RT)

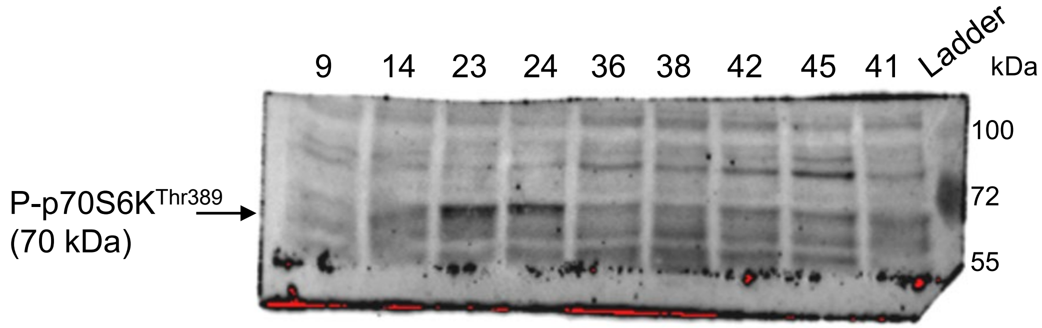

Primary antibody: p70S6kinase (1/1000 in 5% BSA, 1X TBS, 0.1% Tween20 overnight at 4°C)
Secondary antibody: goat anti-rabbit IgG, HRP-linked (1/5000 in 1X TBS, 0.1% Tween20 1h at RT)

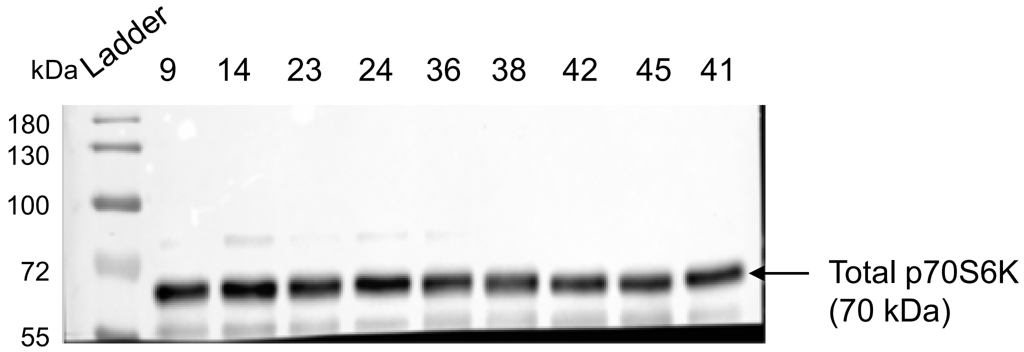

Primary antibody: β-Actin (1/2000 in 5% BSA, 1X TBS, 0.1% Tween20 overnight at 4°C)
Secondary antibody: goat anti-rabbit IgG, HRP-linked (1/5000 in 1X TBS, 0.1% Tween20 1h at RT)

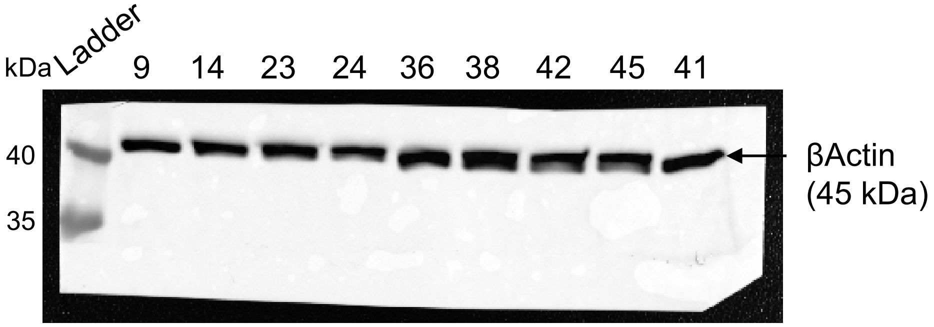

<u>Serie 2</u> : 8 samples/gel, n=2/experimental group
Samples 11, 12, 17, 18, 33, 37, 43, 48

<u>Primary antibody</u>: PI3Kinase p110α (1/1000 in 5% milk, 1X TBS, 0.1% Tween20 overnight at 4°C)
<u>Secondary antibody</u>: goat anti-rabbit IgG, HRP-linked (1/5000 in 1X TBS, 0.1% Tween20 1h at RT)

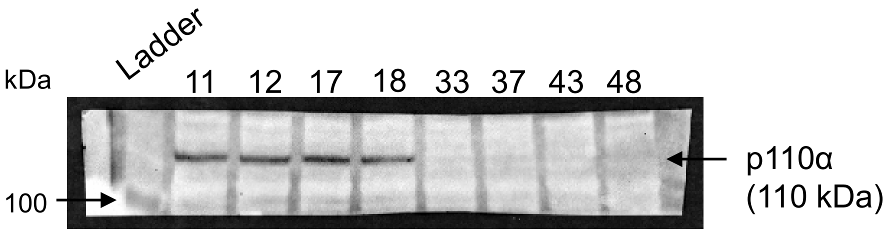

<u>Primary antibody</u>: Phospho-Akt (Ser473) (1/1000 in 5% BSA, 1X TBS, 0.1% Tween20 overnight at 4°C)
<u>Secondary antibody</u>: goat anti-rabbit IgG, HRP-linked (1/5000 in 1X TBS, 0.1% Tween20 1h at RT)

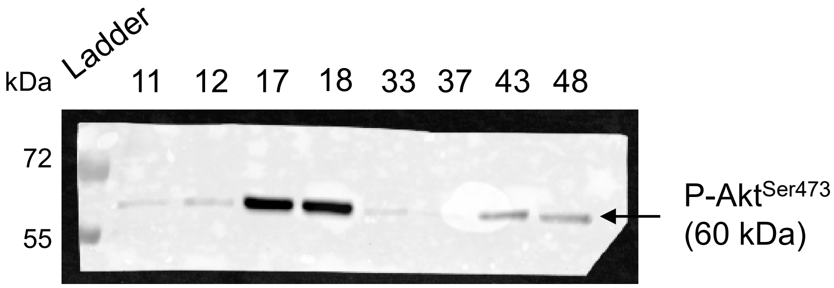

<u>Primary antibody</u>: Phospho-Akt (Thr308) (1/1000 in 5% BSA, 1X TBS, 0.1% Tween20 overnight at 4°C)
<u>Secondary antibody</u>: goat anti-rabbit IgG, HRP-linked (1/5000 in 1X TBS, 0.1% Tween20 1h at RT)

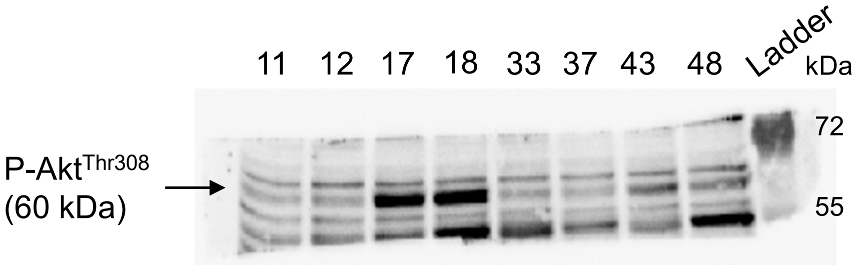

Serie 2 : 8 samples/gel, n=2/experimental group
Samples 11, 12, 17, 18, 33, 37, 43, 48

Primary antibody: Akt (pan) (1/1000 in 5% BSA, 1X TBS, 0.1% Tween20 overnight at 4°C)
Secondary antibody: goat anti-rabbit IgG, HRP-linked (1/5000 in 1X TBS, 0.1% Tween20 1h at RT)

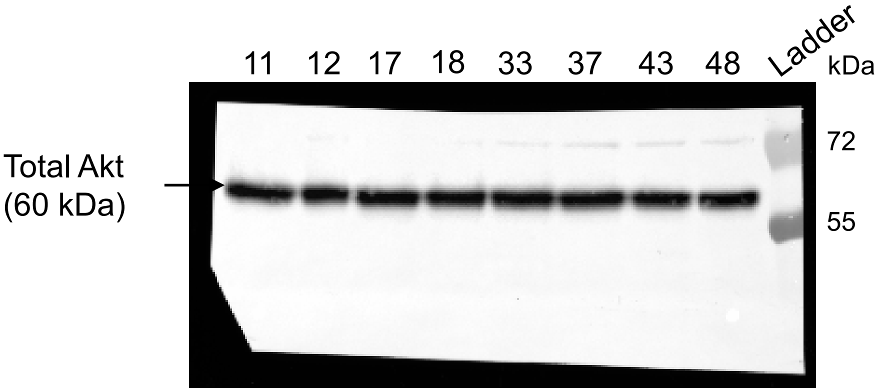

Primary antibody: Phospho- GSK-3β (Ser9) (1/1000 in 5% BSA, 1X TBS, 0.1% Tween20 overnight at 4°C)
Secondary antibody: goat anti-rabbit IgG, HRP-linked (1/5000 in 1X TBS, 0.1% Tween20 1h at RT)

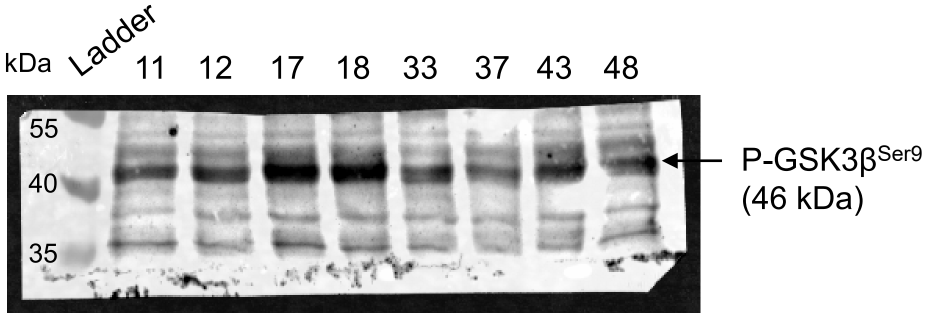

Primary antibody: GSK-3β (1/1000 in 5% BSA, 1X TBS, 0.1% Tween20 overnight at 4°C)
Secondary antibody: goat anti-rabbit IgG, HRP-linked (1/5000 in 1X TBS, 0.1% Tween20 1h at RT)

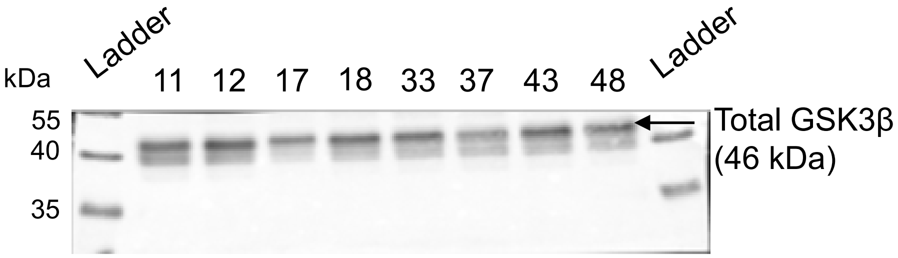

Serie 2 : 8 samples/gel, n=2/experimental group
Samples 11, 12, 17, 18, 33, 37, 43, 48

Primary antibody: Phospho-p70S6kinase (Thr389) (1/1000 in 5% BSA, 1X TBS, 0.1% Tween overnight at 4°C)
Secondary antibody: goat anti-rabbit IgG, HRP-linked (1/5000 in 1X TBS, 0.1% Tween20 1h at RT)

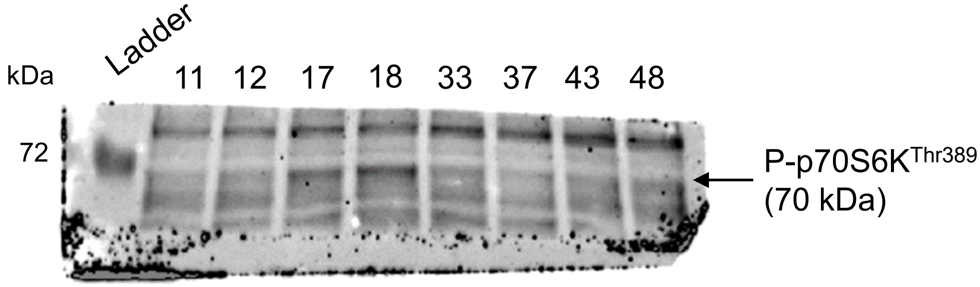

Primary antibody: p70S6kinase (1/1000 in 5% BSA, 1X TBS, 0.1% Tween20 overnight at 4°C)
Secondary antibody: goat anti-rabbit IgG, HRP-linked (1/5000 in 1X TBS, 0.1% Tween20 1h at RT)

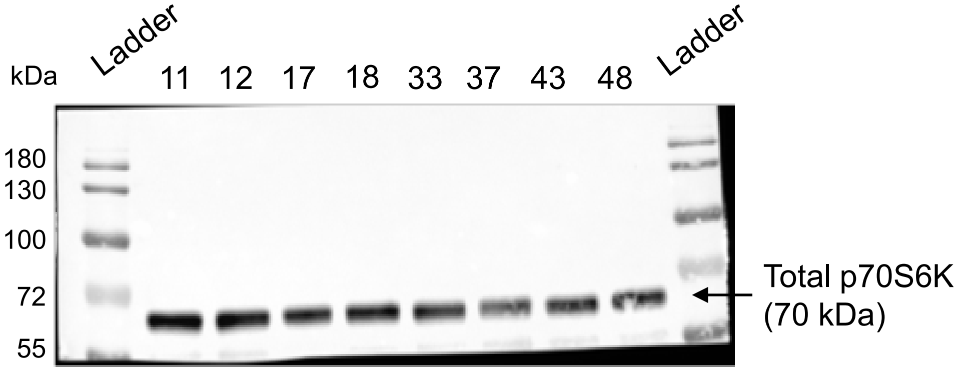

Primary antibody: β-Actin (1/2000 in 5% BSA, 1X TBS, 0.1% Tween20 overnight at 4°C)
Secondary antibody: goat anti-rabbit IgG, HRP-linked (1/5000 in 1X TBS, 0.1% Tween20 1h at RT)

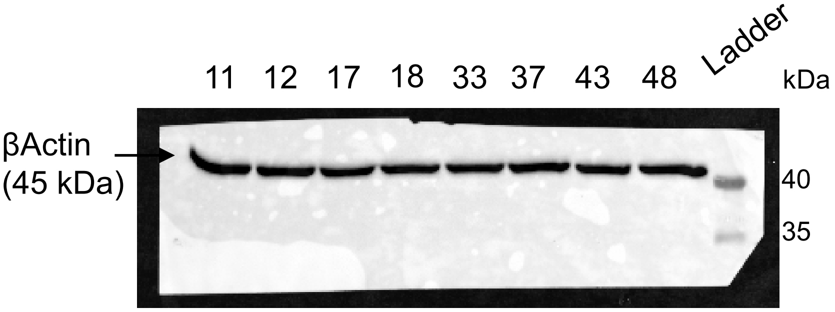

Annotated original images for western blots in Figure 3F

- Serie 1 : 9 samples/gel, n=2-3/experimental group

Samples 23, 24, 28, 30, 50, 51, 57, 58, 60

- Serie 2 : 9 samples/gel, n=2/experimental group

Samples 21, 22, 27, 29, 53, 54, 55, 56, 59

→ In total n=4-6/experimental group

n° sample	genotype	fasted/refed
21	p110α$^{hep+/+}$	fasted
22	p110α$^{hep+/+}$	fasted
23	p110α$^{hep+/+}$	fasted
24	p110α$^{hep+/+}$	fasted
27	p110α$^{hep+/+}$	refed
28	p110α$^{hep+/+}$	refed
29	p110α$^{hep+/+}$	refed
30	p110α$^{hep+/+}$	refed
50	p110α$^{hep-/-}$	fasted
51	p110α$^{hep-/-}$	fasted
53	p110α$^{hep-/-}$	fasted
54	p110α$^{hep-/-}$	fasted
55	p110α$^{hep-/-}$	refed
56	p110α$^{hep-/-}$	refed
57	p110α$^{hep-/-}$	refed
58	p110α$^{hep-/-}$	refed
59	p110α$^{hep-/-}$	refed
60	p110α$^{hep-/-}$	refed

Serie 1 : 9 samples/gel, n=2-3/experimental group
Samples 23, 24, 28, 30, 50, 51, 57, 58, 60

Primary antibody: PI3Kinase p110α (1/1000 in 5% milk, 1X TBS, 0.1% Tween20 overnight at 4°C)
Secondary antibody: goat anti-rabbit IgG, HRP-linked (1/5000 in 1X TBS, 0.1% Tween20 1h at RT)

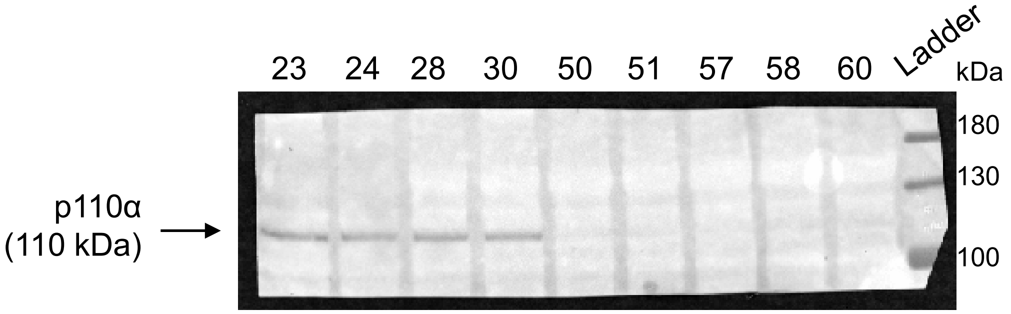

Primary antibody: Phospho-Akt (Ser473) (1/1000 in 5% BSA, 1X TBS, 0.1% Tween20 overnight at 4°C)
Secondary antibody: goat anti-rabbit IgG, HRP-linked (1/5000 in 1X TBS, 0.1% Tween20 1h at RT)

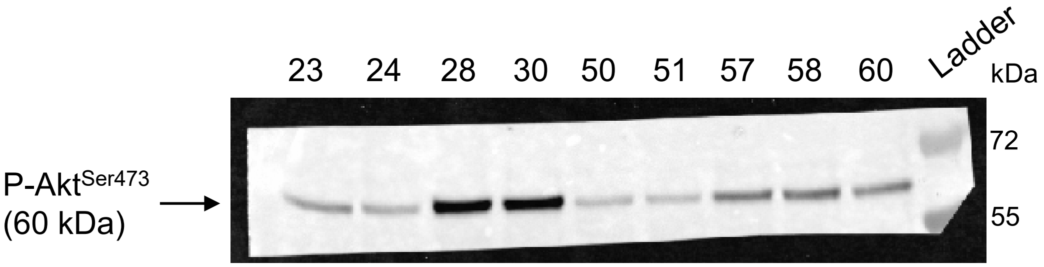

Primary antibody: Phospho-Akt (Thr308) (1/1000 in 5% BSA, 1X TBS, 0.1% Tween20 overnight at 4°C)
Secondary antibody: goat anti-rabbit IgG, HRP-linked (1/5000 in 1X TBS, 0.1% Tween20 1h at RT)

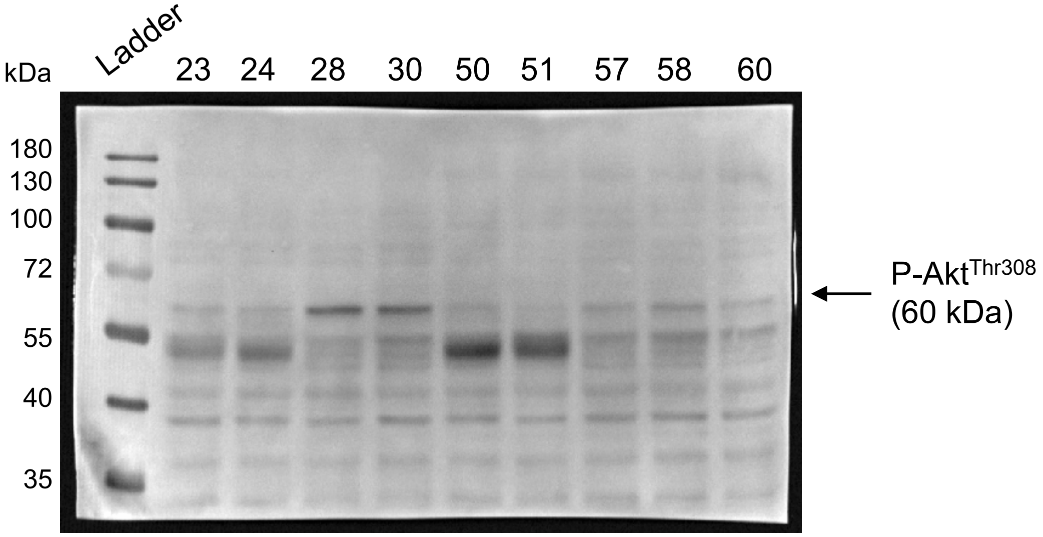

Serie 1 : 9 samples/gel, n=2-3/experimental group
Samples 23, 24, 28, 30, 50, 51, 57, 58, 60

Primary antibody: Akt (pan) (1/1000 in 5% BSA, 1X TBS, 0.1% Tween20 overnight at 4°C)
Secondary antibody: goat anti-rabbit IgG, HRP-linked (1/5000 in 1X TBS, 0.1% Tween20 1h at RT)

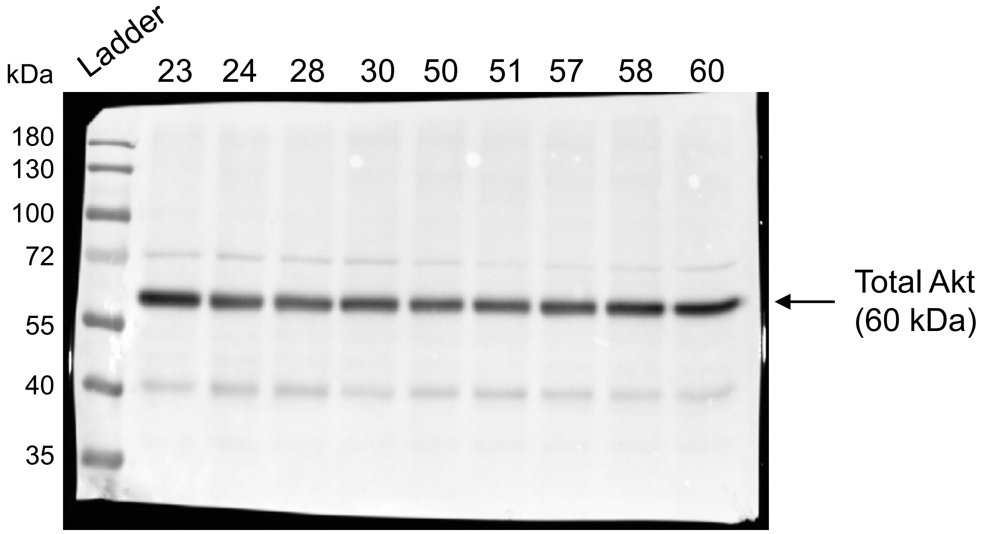

Primary antibody: Phospho- GSK-3β (Ser9) (1/1000 in 5% BSA, 1X TBS, 0.1% Tween20 overnight at 4°C)
Secondary antibody: goat anti-rabbit IgG, HRP-linked (1/5000 in 1X TBS, 0.1% Tween20 1h at RT)

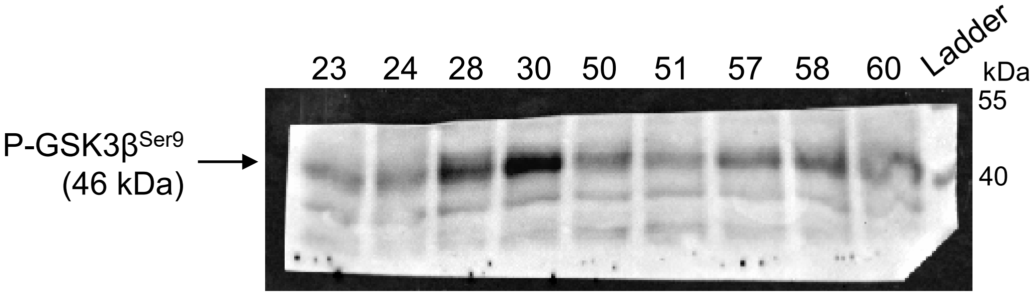

Primary antibody: GSK-3β (1/1000 in 5% BSA, 1X TBS, 0.1% Tween20 overnight at 4°C)
Secondary antibody: goat anti-rabbit IgG, HRP-linked (1/5000 in 1X TBS, 0.1% Tween20 1h at RT)

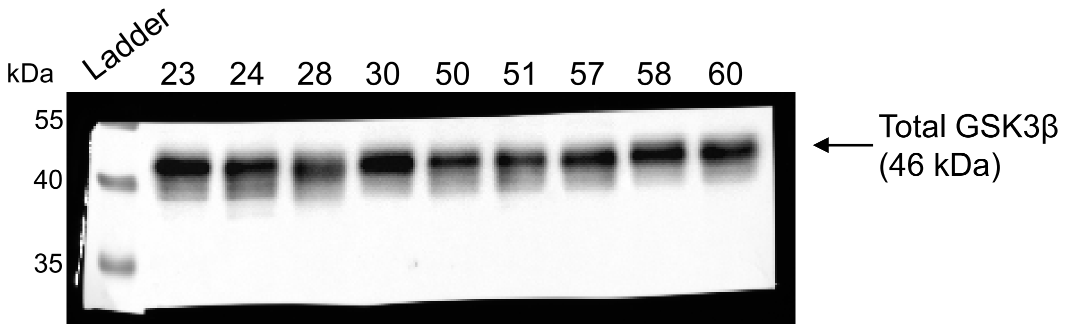

Serie 1 : 9 samples/gel, n=2-3/experimental group
Samples 23, 24, 28, 30, 50, 51, 57, 58, 60

Primary antibody: Phospho-p70S6kinase (Thr389) (1/1000 in 5% BSA, 1X TBS, 0.1% Tween overnight at 4°C)
Secondary antibody: goat anti-rabbit IgG, HRP-linked (1/5000 in 1X TBS, 0.1% Tween20 1h at RT)

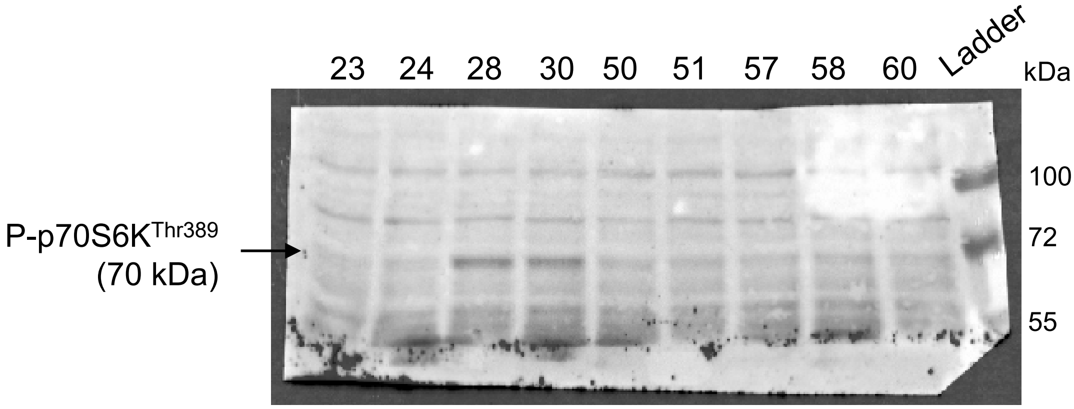

Primary antibody: p70S6kinase (1/1000 in 5% BSA, 1X TBS, 0.1% Tween20 overnight at 4°C)
Secondary antibody: goat anti-rabbit IgG, HRP-linked (1/5000 in 1X TBS, 0.1% Tween20 1h at RT)

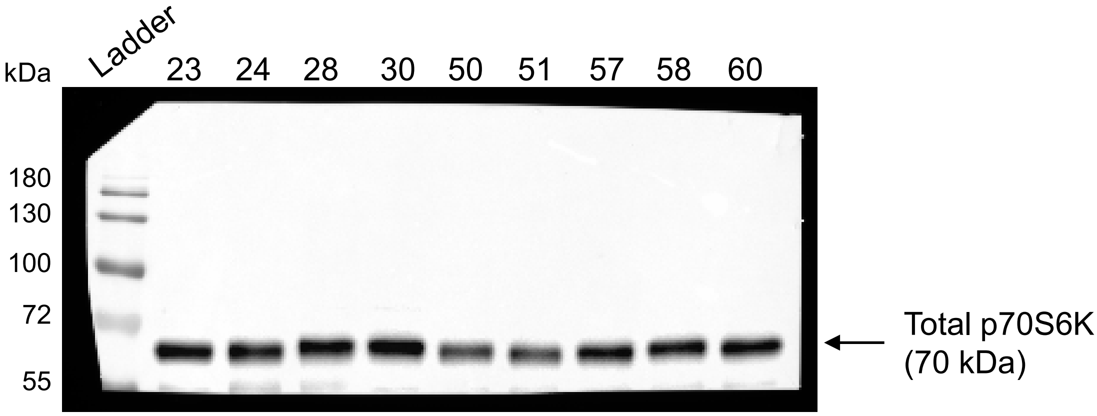

Primary antibody: β-Actin (1/2000 in 5% BSA, 1X TBS, 0.1% Tween20 overnight at 4°C)
Secondary antibody: goat anti-rabbit IgG, HRP-linked (1/5000 in 1X TBS, 0.1% Tween20 1h at RT)

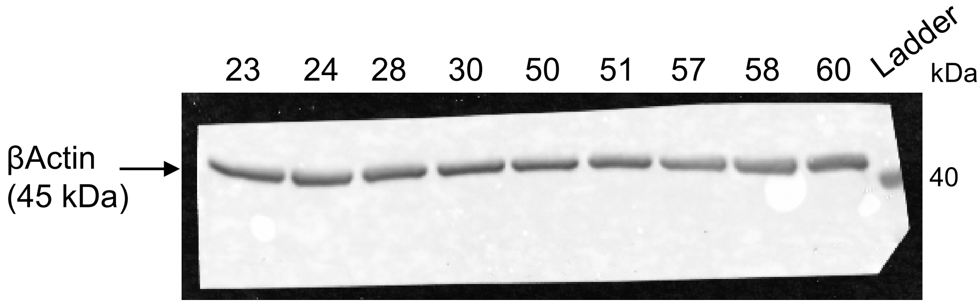

Serie 2 : 9 samples/gel, n=2-3/experimental group
Samples 21, 22, 27, 29, 53, 54, 55, 56, 59

Primary antibody: PI3Kinase p110α (1/1000 in 5% milk, 1X TBS, 0.1% Tween20 overnight at 4°C)
Secondary antibody: goat anti-rabbit IgG, HRP-linked (1/5000 in 1X TBS, 0.1% Tween20 1h at RT)

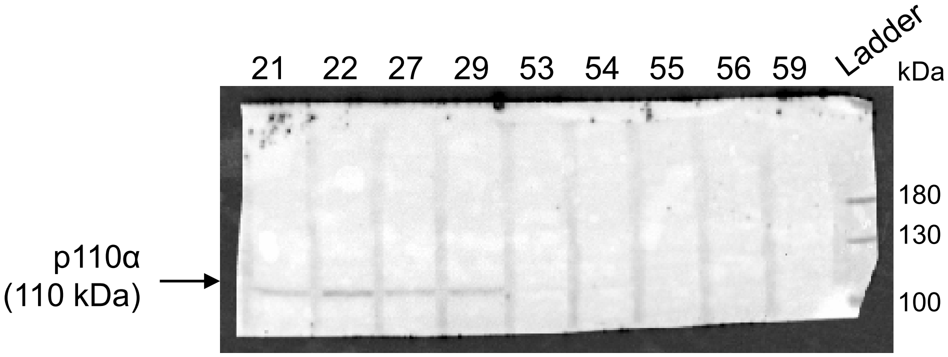

Primary antibody: Phospho-Akt (Ser473) (1/1000 in 5% BSA, 1X TBS, 0.1% Tween20 overnight at 4°C)
Secondary antibody: goat anti-rabbit IgG, HRP-linked (1/5000 in 1X TBS, 0.1% Tween20 1h at RT)

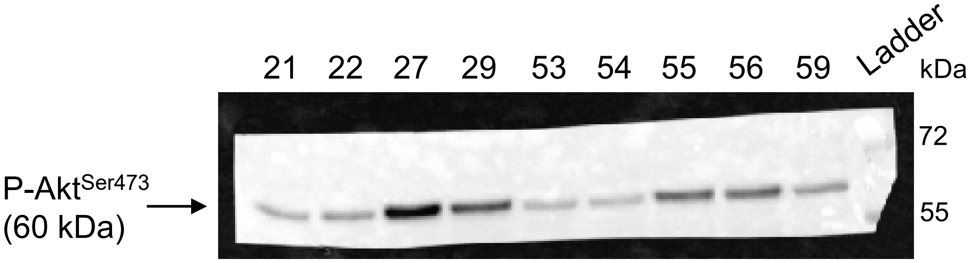

Primary antibody: Phospho-Akt (Thr308) (1/1000 in 5% BSA, 1X TBS, 0.1% Tween20 overnight at 4°C)
Secondary antibody: goat anti-rabbit IgG, HRP-linked (1/5000 in 1X TBS, 0.1% Tween20 1h at RT)

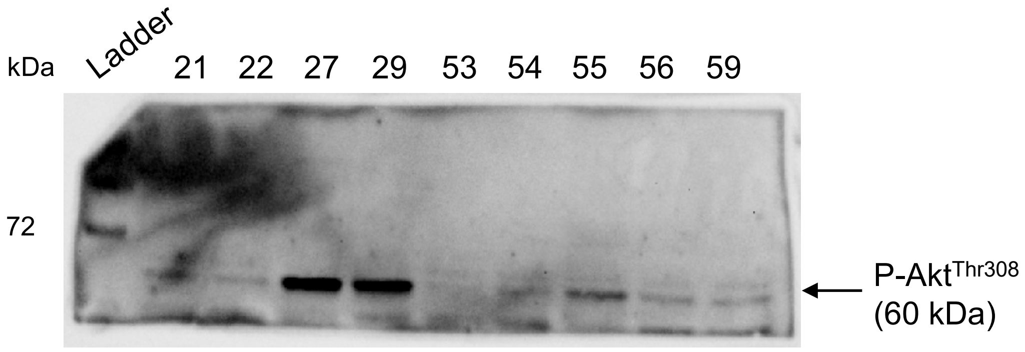

Serie 2 : 9 samples/gel, n=2-3/experimental group
Samples 21, 22, 27, 29, 53, 54, 55, 56, 59

Primary antibody: Akt (pan) (1/1000 in 5% BSA, 1X TBS, 0.1% Tween20 overnight at 4°C)
Secondary antibody: goat anti-rabbit IgG, HRP-linked (1/5000 in 1X TBS, 0.1% Tween20 1h at RT)

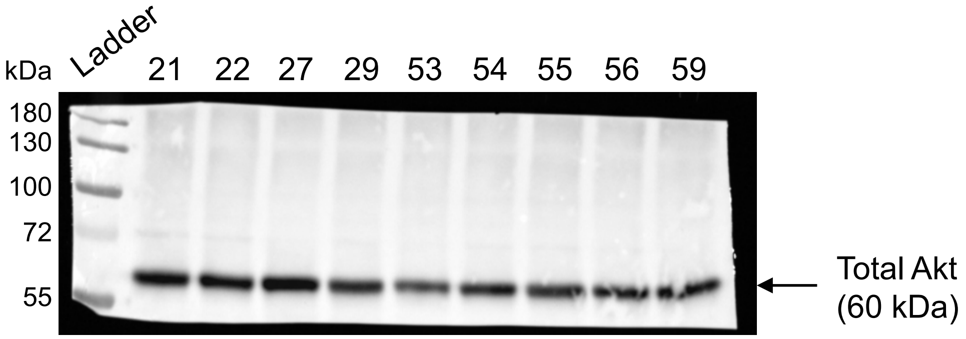

Primary antibody: Phospho- GSK-3β (Ser9) (1/1000 in 5% BSA, 1X TBS, 0.1% Tween20 overnight at 4°C)
Secondary antibody: goat anti-rabbit IgG, HRP-linked (1/5000 in 1X TBS, 0.1% Tween20 1h at RT)

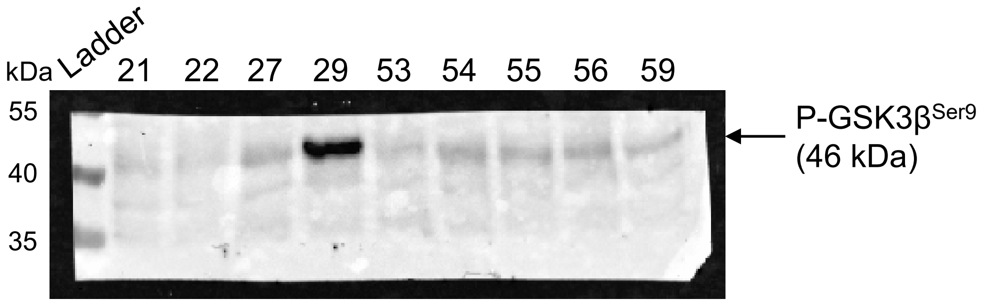

Primary antibody: GSK-3β (1/1000 in 5% BSA, 1X TBS, 0.1% Tween20 overnight at 4°C)
Secondary antibody: goat anti-rabbit IgG, HRP-linked (1/5000 in 1X TBS, 0.1% Tween20 1h at RT)

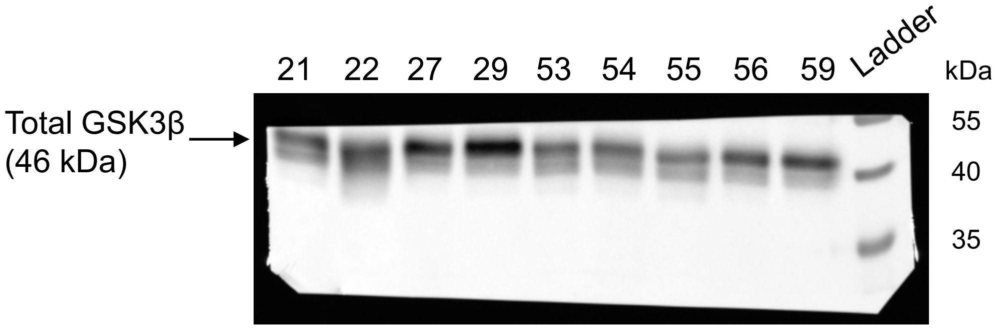

Serie 2 : 9 samples/gel, n=2-3/experimental group
Samples 21, 22, 27, 29, 53, 54, 55, 56, 59

Primary antibody: Phospho-p70S6kinase (Thr389) (1/1000 in 5% BSA, 1X TBS, 0.1% Tween overnight at 4°C)
Secondary antibody: goat anti-rabbit IgG, HRP-linked (1/5000 in 1X TBS, 0.1% Tween20 1h at RT)

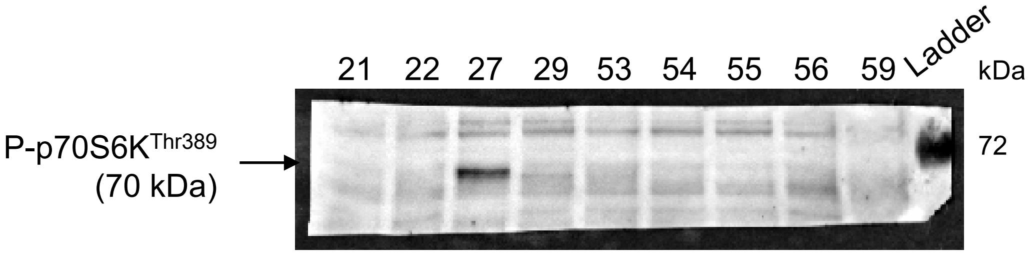

Primary antibody: p70S6kinase (1/1000 in 5% BSA, 1X TBS, 0.1% Tween20 overnight at 4°C)
Secondary antibody: goat anti-rabbit IgG, HRP-linked (1/5000 in 1X TBS, 0.1% Tween20 1h at RT)

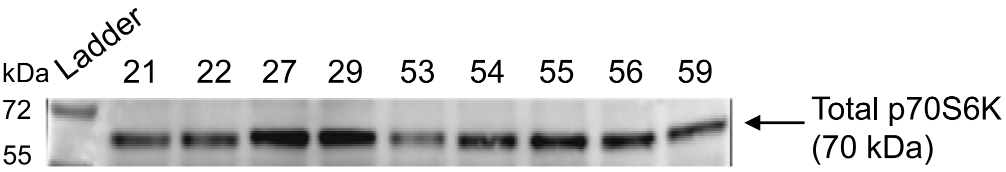

Primary antibody: β-Actin (1/2000 in 5% BSA, 1X TBS, 0.1% Tween20 overnight at 4°C)
Secondary antibody: goat anti-rabbit IgG, HRP-linked (1/5000 in 1X TBS, 0.1% Tween20 1h at RT)

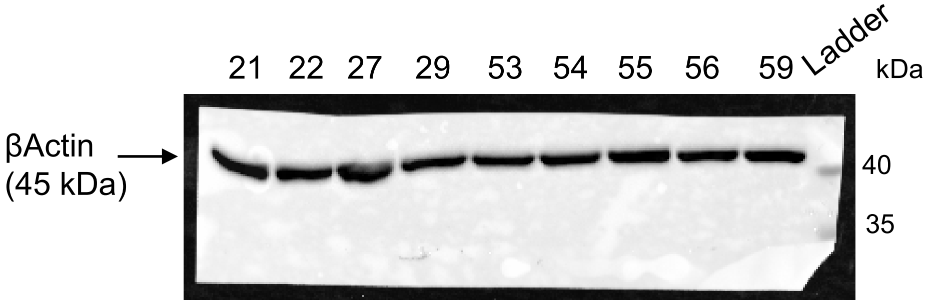

Supplement: S1 Raw Images — (PDF) [file pbio.3003112.s026.pdf]
