# Supplementary material for: Liver gene expression and its rewiring in hepatic steatosis are controlled by PI3Kα-dependent hepatocyte signaling
Source: PLoS Biol. 2025 Apr 14;23(4):e3003112. doi: 10.1371/journal.pbio.3003112 (PMC12021288; doi:10.1371/journal.pbio.3003112)
Supplement: Attachment — Submitted filename: Point by point response.docx [file pbio.3003112.s029.docx]

Thank you for the time you took to review our work. Your fair comments, suggestions and corrections have helped us improve our manuscript.

*Reviewer #1: The metabolic phenotype of liver-specific PI3Kalpha knockout have been previously described (see Ref 16 of this manuscript).*

*The manuscript "p110alpha-dependent hepatocyte signaling is critical for liver gene expression and its rewiring in MASLD" by Marion Régnier et al. investigates the role of PI3Kalpha within the hepatocyte in the control of liver gene expression including the reprogramming induced by HFD, fasting and reefeding, and circadian gene expression. The manuscript is overall interesting but some issue needs to be addressed before publication.*

We thank Reviewer#1 for the constructive feedback and help to improve the current version of our manuscript. We have revised our manuscript taking into consideration all of Reviewer#1 comments that you will find below with our responses.

1) *in the abstract the authors claim that most of PI3K activity induced by insulin is dependent on PI3Kalpha. However, in Figure 1 B the author show that loss of PI3Kalpha in hepatocyte has no effect of baseline PIP3/PIP2 and reduces by 50% the maximal insulin-induced PIP3/PIP2. Therefore it should be concluded that loss of PI3Kalpha has no effect on baseline PI3K activity but it reduces maximal insulin-stimulated PI3K activity.*

Reviewer#1 is right and we made the appropriate correction in the abstract. We claimed that insulin-dependent PI3K activity depend on PI3Kalpha in reference to previously published work. We tried to make this clearer by mentioning the point made by reviewer #1 that PI3Kalpha deletion reduces maximal insulin-induced PI3K activity in the new version of the abstract. We also made similar modification when commenting Fig.1B in the results section.

2) *The immunoblots in Figure 1 C with n=2 are not quantitative. The authors should repeat the blots with at least n=3/4 quantify bands intensities and perform statistical analysis.*

We thank Reviewer#1 for this comment and we made the appropriate repetitions as well as statistical analysis. All the blots have been done at least 4 times. Quantification and statistical analysis are now provided (Supplementary Figure 2B). The analysis reveals a significant reduction of maximal insulin-induced AKT and S6K phosphorylation. In these conditions, the effect on GSK3 phosphorylation was not significant. The text has been modified in the results section.

3) *The data in figure 1E indicate a minor effect of loss of PI3Kalpha in hepatocytes on insulin tolerance. how the authors reconcile this observation with the more pronounced difference in glucose and piruvate tolerance?*

We agree with Reviewer#1. We believe that in the context of a regular diet, these young mice lacking p110alpha in hepatocyte do not show major fasting hyperinsulinemia which would lead to peripheral insulin resistance. The data from Figure 1 likely reflect that p110alpha mice only show a marked defect in the inhibition of hepatic neoglucogenesis by insulin without elevated insulin (Figure 2). Mice lacking p110alpha show defective glucose homeostasis associated with enhanced neoglucogenesis rate. However, despite reduced insulin sensitivity, extra-hepatic tissues remain insulin sensitive.

It was previously shown that deletion of Insulin Receptor (IR) in hepatocytes leads to major hyperinsulinemia which promotes whole body insulin resistance. Unlike IR deletion, p110alpha deletion in hepatocyte only induces a partial defect in insulin signalling. However, hyperinsulinemia and insulin resistance becomes more severe as mice lacking p110alpha in hepatocyte age (as observed in Figure 5).

These important points raised by reviewer#1 are now included in the discussion section of our revised manuscript.

4) *Figure 2 A should include liver glycogen content.*

As suggested by reviewer#1, we performed glycogen content analysis. The method section has been modified to include method used for the glycogen assay. Figure 2A now includes glycogen measurements. The figure legend and the results section have been modified accordingly.

5) *The immunoblots in Figure 3F with n=2 are not quantitative. The authors should repeat the blots with at least n=3/4 quantify bands intensities and perform statistical analysis.*

We thank Reviewer#1 for this comment and we made the appropriate repetitions as well as statistical analysis. All the blots have been done at least 4 times. Quantification and statistical analysis are now provided (Supplementary Figure 4B). The analysis reveal an important variability in phosphorylation levels. The text has been modified in the results section.

6) *Figure 3 should include fasted and refed liver glycogen content.*

As suggested by reviewer#1, we performed glycogen content analysis. Figure 3E now includes glycogen measurements. The figure legend and the results section have been modified accordingly.

7) *Figure 5 should show CTRL and HFD liver glycogen content ideally over 4 ZT time points covering the 24 hrs liver glycogen rhythm.*

As suggested by reviewer#1, we performed glycogen content analysis over 5 ZT time points. Supplemental Figure 10 present these new glycogen measurements. Supplemental Figure 10 legend and result section have been modified accordingly. Because glycogen measurements have been analysed for their circadian rhythmicity , we chose to introduce them in the results section corresponding to Figure 7 and circadian analysis.

Altogether, the glycogen measurements we have done across the different experimental settings suggest that PI3K signalling dependant on p110alpha is largely dispensable for glycogen synthesis in response to feeding. These new results are now discussed in this revised version of the manuscript. We thank reviewer#1 for this interesting suggestion.

*Reviewer #2: The manuscript by Régnier et al. reports phenotypes of liver-specific deletion of PI3Ka upon HFD and other feeding-induced metabolic challenges.*

*The phenotypic effects of liver-specific deletion of PI3Kα—such as the development of insulin resistance and protection from liver steatosis upon high-fat diet (HFD) feeding—have been reported in several publications, which are cited in the current manuscript. Nevertheless, the study under review has significant merit. It presents a comprehensive transcriptomic analysis of PI3K's role in regulating liver gene expression, comparing and contrasting the effects of PI3Kα deletion with those of insulin receptor deletion. These findings are further supported by an extensive integration of metabolite profiling data with the gene expression results.*

*Among its key findings, the study demonstrates that PI3Kα does not regulate ChREBP or PPARα but is involved in the regulation of CREBH, intriguingly in an insulin receptor-independent manner. This represents a novel and noteworthy discovery. Additionally, the study implicates PI3Kα in the regulation of rhythmic gene expression in the liver.*

*Overall, these data provide a meaningful contribution to the field of insulin signaling research, offering deeper insights into the role of PI3K in metabolic regulation. While the study is generally well-presented, a few areas require clarification and improvement. Also, additional proofreading is needed to enhance figure legends and labeling for greater clarity.*

We thank Reviewer#2 for the constructive feedback and help to improve the current version of our manuscript. We have revised our manuscript taking into consideration Reviewer#2 comments that you will find below with our responses.

*In terms of criticisms, a limitation of the gene-targeted mouse model used in this study, which is effectively a conditional knockout, is that such models can exhibit partial functional compensation by other isoforms when studying pathways with multiple isoforms, such as the PI3K pathway. The ability of another isoform to compensate in an artificial system where the targeted isoform (PI3Kα in this case) is physically absent (as in a knockout) does not necessarily imply that the compensating isoform could fulfill the same role physiologically in a normal cell, where the PI3Kα isoform is intact. Consequently, conditional knockout models may not accurately represent the physiological function of the targeted protein compared to catalytically inactive point mutants or treatments with isoform-selective inhibitors. Unfortunately, many studies utilizing conditional knockout PI3K models fail to acknowledge this limitation, detracting from their scientific precision.*

We thank Reviewer#2 for this fair criticism. We thought we could take it into consideration by adding a section related to this limitation of the study in the discussion section.

Therefore, the following section was added to the conclusion in the discussion: “One limitation of our study is that we used a mouse model with conditional deletion of p110α, the catalytic subunit of PI3Kα. This may have led to compensation by other PI3K isotypes such as p110β, which is also expressed in hepatocytes, and may have led us to underestimate the role of p110α in hepatocytes. Further studies are needed to assess the specific roles of PI3K isotypes in liver health and in metabolic diseases.”

*Discussion Section: The statement, "Moreover, the present study suggests that hepatocyte p110α selectively phosphorylates stearoyl/arachidonoyl species of PIP2 to produce PIP3 in response to insulin," likely refers to the phosphoinositide profiling shown in Supplementary Figure 2. However, the legend for this figure lacks sufficient detail to clarify the designations in the graphs. Furthermore, the interpretation of these data in the corresponding sections of the main text is minimal and would benefit from further elaboration.*

We thank Reviewer#2 for the constructive suggestions. We now provide more details to clarify the designations of the graphs in Supplementary Figure 2. The legend now states that the analysis was performed by mass spectrometry and the relative abundance for each molecular species were calculated as a ratio to Phosphatidylinositol (PI). This method yields the number of phosphorylations and the fatty-acyl compositions of phosphatidylinositol. For example, PIP3 (C38:4) means that the phosphatidylinositol lipid has a mass that corresponds to three phosphorylations of the inositol and contains acyl chains with 38 carbons and 4 double bonds. This is the only PIP3 (C38:4) that is detectable and therefore we cannot conclude about possible selective substrate specificity of PI3Kα. Therefore, we deleted our misleading sentence in the discussion. We also agree that our interpretation was minimal and we provided an additional sentence in the corresponding result section.

Minor issues:

*Main text cites 'Fig 8' instead of the correct 'Fig. 7'*

Sorry about this. The appropriate corrections have been made through the main text.

*Fig 7A statistical significance comparisons is unclear.*

Sorry about this. The appropriate addition was made to Figure 7A legend. Data are presented as mean ± SEM. ^#^P≤0.05 and ^###^P≤0.005 for diet effect; *P≤0.05 and ***P≤0.005 for genotype effect.

*Fig 7H legend: It is not clear what is meant by "rows" and "columns"*

Thank you for pointing at this mistake. We made the appropriate corrections in Figure 7G and Figure 7H legend.

*Suppl Fig. 5 lacking explanation of statistical comparisons*

Sorry about this. We have made the appropriate additions to Supplementary Figure 5 legend.

*Suppl Fig. 6 panel C lacking a legend description*

Sorry about this. We have made the appropriate additions to Supplementary Figure 6 legend.
